# Mathematical Models of the Impact of IL2 Modulation Therapies on T Cell Dynamics

**DOI:** 10.3389/fimmu.2013.00439

**Published:** 2013-12-11

**Authors:** Kalet León, Karina García-Martínez, Tania Carmenate

**Affiliations:** ^1^Systems Biology Department, Center of Molecular Immunology, Habana, Cuba

**Keywords:** mathematical model, T cell dynamics, interleukin 2, interleukin 2 mutants, regulatory T cells

## Abstract

Several reports in the literature have drawn a complex picture of the effect of treatments aiming to modulate IL2 activity *in vivo*. They seem to promote either immunity or tolerance, probably depending on the specific context, dose, and timing of their application. Such complexity might derive from the pleiotropic role of IL2 in T cell dynamics. To theoretically address the latter possibility, our group has developed several mathematical models for Helper, Regulatory, and Memory T cell population dynamics, which account for most well-known facts concerning their relationship with IL2. We have simulated the effect of several types of therapies, including the injection of: IL2; antibodies anti-IL2; IL2/anti-IL2 immune-complexes; and mutant variants of IL2. We studied the qualitative and quantitative conditions of dose and timing for these treatments which allow them to potentiate either immunity or tolerance. Our results provide reasonable explanations for the existent pre-clinical and clinical data, predict some novel treatments, and further provide interesting practical guidelines to optimize the future application of these types of treatments.

## Introduction

Several reports in the literature have drawn a complex picture of the effect of treatments aiming to modulate IL2 activity *in vivo*. These treatments seem to promote either immunity or tolerance, probably depending on the specific context, dose, and timing of their application.

Treatments that increase IL2 activity, simply by injecting it, have been shown to potentiate the immune response to vaccines (1–4) and are a current medical practice to enhance the natural anti-tumor immunity in patients with melanoma. However, several reports in the literature have shown that HIV (5–8) and melanoma (9) patients treated with IL2, experience an increase in CD4^+^ CD25^+^ FoxP3^+^ regulatory T cells, which typically mediate natural immune tolerance. Moreover, several pre-clinical studies have further documented a tolerogenic effect of IL2. Injections of IL2 have been shown to prevent or ameliorate autoimmune responses in mice (10–12). Treatments which reduce natural IL2 activity, by sequestering it with anti-IL2 monoclonal antibodies, have been shown to induce autoimmune responses (13). And treatments intending to block IL2 activity, with non-depleting anti-IL2-receptor antibodies, are showed to have anti-tumoral effects (14). Nevertheless, in the clinical practice non-depleting anti-IL2-receptor antibodies are used to ameliorate the autoimmune reaction in patients with neoplasia, autoimmune diseases, and organ allograft rejection (15).

Further complexity to the latter picture has been recently added with the pre-clinical assessment of treatments based on immune-complexes formed by IL2 and monoclonal antibodies anti-IL2. This treatment shows a much more potent *in vivo* effect than IL2 alone, appears again to potentiate either immunity (16, 17) or tolerance (18), depending on the specific antibody used to form the immune-complexes. In particular, the specific epitope in the IL2 recognized by the antibody has been postulated as critical for this phenomenon (19, 20).

IL2 interacts with many different cells types, which express the three known chains of the IL2 receptor. Particularly relevant and complex is its relationship with the population dynamics of the CD4 T lymphocytes. IL2 was originally described as a potent CD4^+^ T cell growth factor (21), which should in consequence enhance overall T cell immunity. However, several experiments have shown lately a critical role for this cytokine in the survival and proliferation of the CD4^+^CD25^+^FoxP3^+^ T cells (regulatory T cells) (22, 23), which mediate the maintenance of natural and induced tolerance. The CD4^+^CD25^−^FoxP3^−^ T cells (helper T cells) have been identified as the principal source of IL2 *in vivo* (24), suggesting that the regulatory T cells have to sequester the IL2 produced by these cells in order to proliferate and survive (25). Moreover, *in vitro and in vivo* experiments have shown that regulatory T cells inhibit the production of IL2 by the helper T cells (26), limiting in this way their own source of this essential cytokine. Thus, overall, it seems that IL2 has a dual role on its circuit of interactions with CD4^+^ T cells. It could promote the proliferation of the helper T cells, which may drive effective immunity and foster IL2 production. But, it could also promote the expansion of regulatory T cells, which may turn off the immune reaction, as well as the IL2 production on its own. The dynamic balance between these opposite forces might explain the complexity observed in the effect of treatments that modulate IL2 activity, either sequestering it or further increasing it.

To theoretically address the latter hypothesis, our group has developed mathematical models for Helper, Regulatory, and Memory T cells dynamics, which account for most well-known facts relative to their relationship with IL2. We have simulated the effect of several types of therapies including the injection of: IL2; antibodies anti-IL2; IL2/anti-IL2 immune-complexes, and mutants variants of IL2. We studied the qualitative and quantitative conditions of dose and timing for these treatments which allow them to potentiate either immunity or tolerance. Our results provide reasonable explanations for the existent pre-clinical and clinical data, predict some novel treatments, and further provide interesting practical guidelines to optimize the future application of these types of treatments.

## Materials and Methods

### Introduction to the mathematical model

**Figure 1:**
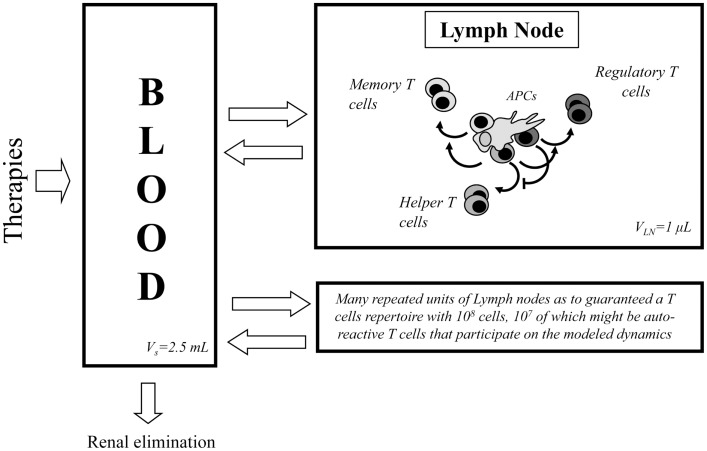
**Diagram of the processes occurring in the two compartments considered in the model**. At the left side of the diagram the blood compartment is shown, where soluble molecules related with IL2 modulatory therapies are introduced and eliminated. This compartment is in constant molecular exchange with the lymph nodes (right side of the diagram). In this last class of compartment, occur the processes related with the dynamics of T cells and their interaction with the IL2 and other soluble molecules.

The mathematical model used in this paper is based on the one developed in Ref. (27) to describe the interaction between IL2 and helper (E) and regulatory (R) CD4^+^ T cells and memory CD8^+^ T cells inside a lymph node. The model includes several physical compartments, which minimally capture the bio-distribution of T cells, IL2, and antibodies in the immune system (see Figure 1). It includes several compartments, which represent different lymph nodes, where T cells are confined interacting with each other’s, with the antigen presenting cells (APCs) and available soluble molecules. It includes also a compartment representing the blood (i.e., the circulatory system), which contains only soluble molecules, IL2, mutant variants of IL2 or anti-IL2 antibodies. Each lymph node in the system is connected to the blood compartment, allowing the free exchange of these soluble molecules.

### Dynamics in the blood compartment

The concentration of soluble molecules in the blood compartment is assumed to decay with a constant characteristic rate, which represent renal elimination in the kidney. An external source term for these molecules is added in this compartment to simulate particular treatment applications. Interaction between free IL2 and anti-IL2 antibodies are modeled in this and other compartments as a dynamic equilibrium characterized by a given biding affinity. Equations for the dynamics in this compartment are presented in “Dynamics in the Blood Compartment” in Appendix A.

### Dynamics for T cells inside lymph nodes

**Figure 2:**
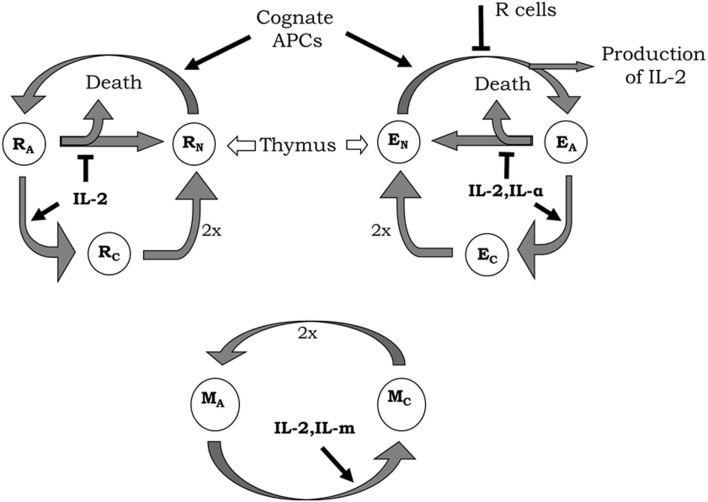
**Diagrams of helper (E), regulatory (R), and memory (M) T-cell life cycle considered in the model**. New resting E (E_N_) and R (R_N_) cells are constantly generated by the thymus. These resting T cells become activated by interaction with their cognate APCs. During activation, E cells produce IL2, although the whole process can be inhibited by the presence of co-localized R cells. Activated E (E_A_) and R (R_A_) enter the cell cycle (becoming cycling cells) when receiving enough signal from IL2 or another external cytokine (IL-α) in the case of E cells. In the absence of enough cytokines, activated T cells become inactivated, where a fraction of cells simply returns back to the resting state and the other dies. Cycling E (E_C_) and R (R_C_) cells divide with a constant rate generating two new resting E or R cells, respectively. Memory T cells are assumed as being always in a sort of naturally activated state (even without any strong cognate interaction with APCs). Activated M (M_A_) cells enter the cell cycle when receiving enough signals from IL2 or another external cytokine (IL-m). Cycling M cells (M_C_) divide generating two new activated M cells.

The model includes, inside the lymph nodes, the dynamics of Helper (E), and Regulatory (R) T cells on three different functional states of their life cycle: resting, activated, and cycling cells. All the interactions involving these T cells occur in the presence of a constant amount of their cognate APCs and relevant homeostatic cytokines. The basic processes and interactions included in the model dynamics for these T cells are (see Figure 2 and (27, 28) for a more detailed biological explanation, including references to experiments that sustained their validity):

i. Resting E and R cells are produced at constant rate by the thymus; they die with a constant decay rate; they get activated (becoming an activated cell) following conjugation to their cognate APCs. The activation of E cells can be inhibited by the presence of co-localized R cells on the APCs.
ii. The activated E and R cells could become cycling cells following a dose-dependent response to cytokine derived signals. The activated R cells get this signal from the interaction with available IL2 while the E cells could additionally use other homeostatic cytokines^1^, which are referred in the model as ILa and are available inside the lymph node in a constant but limited amount. In the absence of enough cytokine derived signal, a fraction of the activated E or R cells revert to the resting state and the remaining fraction just die.
iii. The cycling E and R cells are fully committed to divide, producing two new resting cells. Thus, they are presumed to do so with a constant rate.

The model includes also the dynamics of a generic population of non-CD4 T cells, which binds weakly to the existent APCs, but proliferates in response to IL2 signal, with similar sensitivity than the activated helper CD4^+^ T cells. This cells (referred as M cells) represent, the memory CD8^+^CD44^+^ T cells, which can proliferate in response to IL2 without any requirements of activation by cognate APCs (see Figure 2).

The dynamics of the number of T cells in the lymph node compartment, following the process described above, are modeled with the set of equations presented in “Dynamics of T Cells in the Lymph Node Compartment” in Appendix B.

### Dynamics IL2 and antibodies anti-IL2 inside the lymph node

**Figure 3:**
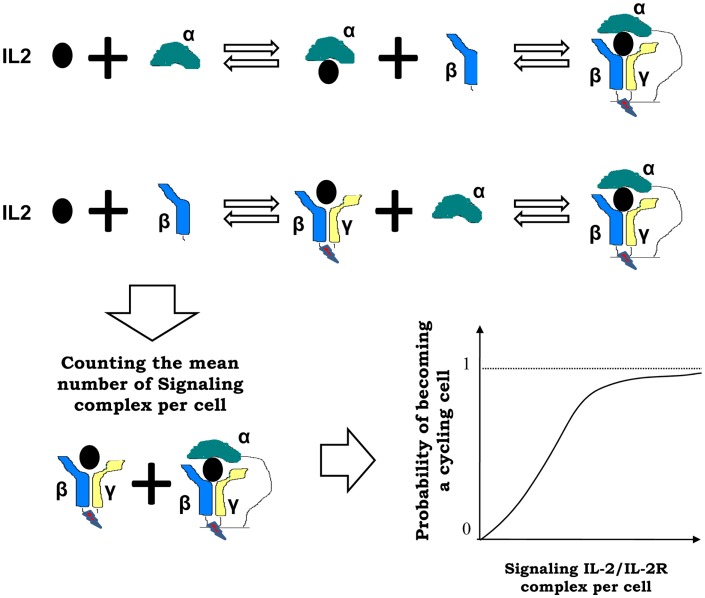
**Interactions between IL2 and T cells in the model are mediated by the IL2 receptor (IL2R), which is formed by three chains: alpha, beta, and gamma chain**. These chains are combined dynamically in multi-step process at the cell surface, upon IL2 binding, to conform the two known signaling forms of the IL2 receptors: high affinity alpha-beta-gamma and intermediate affinity beta-gamma receptor. In the model, the mean number of such signaling IL2-IL2R complexes per activated T cell are counted, and the probability of becoming a cycling cell is computed with a sigmoid function of the mean number of bound cytokines signaling receptors per cell (as shown at the right side of the arrow).

The dynamics of IL2 molecules inside the lymph node takes into account the role of T cells in the production and degradation of this cytokine. The following processes are considered in the model [see Figure 2 and Ref. (27) for a more detailed biological explanation, including references to experiments that sustained their validity]:

iv. IL2 is produced by E cells upon activation. It is produced as a burst whenever a resting E cell becomes an activated E cell. Such production of IL2 is inhibited, together with the E cell activation, by the presence of co-localized R cells on the APCs.
v. IL2 is degraded in the lymph nodes, after being internalized by the T cells in the form of complexes with the IL2 receptor at their cell surface.
  Interactions of IL2 and T cells in the model are based on the expression by these cells, either in the resting, activated or cycling state, of different levels of the IL2 receptor. These receptors mediate the binding of IL2, which provide a stimulatory signal in a dose-dependent fashion to the T cell. In this model the three known chains of the IL2 receptor, alpha, beta, and gamma (29) are included. These three chains are combined dynamically at the cell surface, upon IL2 binding, to conform the two known signaling forms of the IL2 receptors. The following processes and known facts are considered in the model regarding this interaction [see Figure 3 and Ref. (27)]:
vi. IL2/IL2Receptor complexes formation is modeled as a multi-step process: free, soluble, IL2 binds initially to the available free alpha or free beta chains of the receptor, and only then can form dimers or trimers with the remaining IL2 receptor chains at the cell membrane. The gamma chain is assumed to be always in excess compared with the amount of beta chain bound to IL2, either alone or together with alpha chain. Therefore gamma chain joins immediately to these membrane complexes, forming the well known intermediate (beta-gamma-IL2) or high affinity (alpha-beta-gamma-IL2) IL2–IL2 receptor complexes.
vii. IL2/IL2Receptor configurations, which include the beta and gamma chains (high-affinity alpha-beta-gamma, and intermediate affinity beta-gamma receptor), trigger a signal into the T cells (19). Therefore, in the model, the mean number of such signaling receptors per activated E cell, R cell, and M cell are counted. Then, the probability of getting enough signal as to become a cycling cell, for any particular activated E, R, or M cell, is computed with a sigmoid dose response curve, of the mean signaling level. The use of a sigmoid dose response curve is based on direct experimental observations on *in vitro* culture of CD4^+^ T cells (30) stimulated with recombinant IL2.
viii. Beta and gamma chain of the IL2 receptor are similarly expressed by E and R cells in all functional states, but the expression of the alpha chain is modulated with T cell activation (31). R cells constitutively express the alpha chain in the resting state, but further increase its expression level with activation. E cells do not express the alpha chain in the resting state, but gain a significant expression level with activation.
ix. The M cells are assumed to express a negligible amount of the alpha chain of IL2 receptor, but have levels of the beta and gamma chain which are higher than those of helper and regulatory T cells (32).

Antibodies anti-IL2 are modeled as molecules that can form complexes with the IL2, blocking or not its binding to the different chains of the IL2 receptor at the T cell surface. IL-2 mutants are modeled as a molecule bearing similar properties than wild-type IL-2, but differing in some specific parameter value on each case. In particular, we simulate the effects of IL2 mutants with an either reduced or increased Kon for the alpha or beta chains of the IL2R.

The equations in the model describing the dynamics of the number of molecules circulating in the Lymph Node (IL2, anti-IL2 antibodies, and immune-complexes) and the number of complexes IL2-IL2R and IL2-mAb-IL2R formed in a single cell membrane are described in “Dynamics of Molecules in the Lymph Node” in Appendix C.

### Simulation of different therapies

Four types of treatments are simulated in the model: injections of IL2; injections of anti-IL2 monoclonal antibodies; injections of immune complex composed of a mixture of IL2 and anti-IL2 antibodies with a specified constant proportion of them; and injection of mutant variants of IL2.

Treatments are simulated to represent a continuous infusion of the involved molecules for a defined period of time. This is implemented by setting on, transiently, the external source term of the molecules involved in a specific treatment (i.e., IL2; IL2m; and/or anti-IL2 antibody). Two parameters always control treatment application: the “dose,” which set up the total amount per day of IL2, IL2m, and/or anti-IL2 antibody infused; and the “treatment duration,” which set the time period for which continuous infusion is maintained. In all cases, we explore how the dose and treatment duration determine the outcome of the system simulation. We study whether or not different treatments can condition a significant preferential expansion (dominance) of helper T cells or regulatory T cells or M cells in the system.

### Parameter and variable values in model simulations

Model parameters were previously calibrated in Ref. (27). The actual values of parameter used in our simulations are provided in Tables 1–3. The majority of the model parameters are fixed to values directly taken or derived from available independent experimental data; just a few parameters remain unknown, and their influence in result was explored inside a range of biologically reasonable values. Given the realistic values and units of the most model parameters used in the simulations, we report in this paper the values of treatments doses in milligrams and the values of treatment duration in weeks. However, the reader should note that our model is only roughly calibrated, thus one should believe on the order of magnitude and general qualitative trends of the predicted effects. But, the exact values of dose and treatment duration reported here to cause a given effect in the simulations should not be taken as a solid prediction.

**Table 1:** **Variables and parameters appearing in the equations that model the dynamics in the blood compartment**.

Variables	Definitions	
IL2_S_	Total number of IL2 molecules in the blood	
IL2m_S_	Total number of IL2m molecules in the blood	
Ab_S_	Total number of anti-IL2 mAb in the blood	
${\text{IL2}}_{\text{S}}^{\text{Ab}}$	Total number of IL2-mAb complexes in the blood	
IL2	Total number of free IL2 molecules (non-conjugated to IL2R at the cell membrane) in the lymph node	
IL2m	Total number of free IL2m molecules (non-conjugated to IL2R at the cell membrane) in the lymph node	
Ab	Total number of free anti-IL2 mAb (non-conjugated to IL2-IL2R complex at the cell membrane) in the lymph node	
IL2^Ab^	Total number of free IL2-mAb complexes (non-conjugated to IL2R at the cell membrane) in the lymph node	

**Symbolic labels**	**Definitions**	

*j*	Symbolic label that denotes the different IL2R chains: *j* = *α* (alpha chain) and *j* = *β* (beta chain)	
*l*	Symbolic label that denotes the possible functional states of the T cells: *l* = *N* resting state, *l* = *A* activated state and *l* = *C* cycling state	

**Parameters**	**Definitions**	**Values used in simulations**

Γ*_i_*	External influx of IL2, typically used to simulate IL2 addition treatment	*–*
*K*_pi_	Rate of IL2 production by helper CD4^+^ T cells upon activation	10^3^ M/h
*K*_di_	Elimination rate of IL2 in the blood	Ln(2)/10 min
*N*_LN_	Total number of equivalent lymph nodes considered in the system	10
*D*_il_, *D*_ab_	Diffusion rate for the exchange of IL2 and mAbs, between the blood and peripheral lymph nodes	10^−7^ L ×Ln(2)/10 min
*V*_S_, *V*_LN_	Volume of the blood and lymph node compartments, respectively	2.5 × 10^−3^ L, 10^−6^ L
fve	Fraction of the lymph node volume, in which molecules and mAbs can diffuse	0.1
$K_{on}^{Ab},K_{off}^{Ab}$	Association and dissociation constants of IL2-mAb complexes	Face alpha mAb: 1.5 × 10^5^ M^−1^s^−1^, 1.4 × 10^−4^ s^−1^; face beta mAb: 2.3 × 10^4^ M^−1^s^−1^, 6.6 × 10^−5^s^−1^
Γ_mi_	External influx of IL2m, typically used to simulate IL2 addition treatment	*–*
Γ_ab_	External influx of mAb, typically used to simulate anti-IL2 mAbs addition treatment	*–*
K_da_	Elimination rate of mAbs and IL2-mAbs complexes in the blood	Ln(2)/3 days
*N*_A_	Avogadro’s number	6,02 × 10^23^ mol^−1^

**Table 2:** **Variables and parameters appearing in the equations that model the T cells dynamics**.

Variables	Definitions	
*E*_N_, *E*_A_, *E*_C_	Total number (conjugated plus non-conjugated) of resting, activated, and cycling E cells	
*R*_N_, *R*_A_, *R*_C_	Total number (conjugated plus non-conjugated) of resting, activated, and cycling R cells	
*M*_A_, *M*_C_	Total number (conjugated plus non-conjugated) of activated and cycling M cells	

**Intermediate variables**	**Definitions**	

$E_{\text{N}}^{\text{B}},\;E_{\text{A}}^{\text{B}},\;E_{\text{C}}^{\text{B}}$	Number of resting, activated, and cycling E cells conjugated to APCs	
$E_{\text{T}}^{\text{B}}$	Total number of conjugated E cells: $E_{\text{T}}^{\text{B}}=E_{\text{N}}^{\text{B}}+E_{\text{A}}^{\text{B}}+E_{\text{C}}^{\text{B}}$	
$E_{\text{N}}^{\text{F}},\;E_{\text{A}}^{\text{F}},\;E_{\text{C}}^{\text{F}}$	Number of resting, activated, and cycling E cells non-conjugated to APCs: $E_{\text{l}}^{\text{F}}=E_{l}-E_{\text{l}}^{\text{B}},\;\;\forall l\in \left\{N,\;A,\;C\right\}$	
$R_{\text{N}}^{\text{B}},\;R_{\text{A}}^{\text{B}},\;R_{\text{C}}^{\text{B}}$	Number of resting, activated, and cycling R cells conjugated to APCs	
$R_{\text{T}}^{\text{B}}$	Total number of conjugated R cells: $R_{\text{T}}^{\text{B}}=R_{\text{N}}^{\text{B}}+R_{\text{A}}^{\text{B}}+R_{\text{C}}^{\text{B}}$	
$R_{\text{N}}^{\text{F}},R_{\text{A}}^{\text{F}},R_{\text{C}}^{\text{F}}$	Number of resting, activated and cycling R cells non-conjugated to APCs: $R_{\text{l}}^{\text{F}}=R_{l}-R_{\text{l}}^{\text{B}},\;\forall l\in \left\{N,\;A,\;C\right\}$	
$M_{\text{A}}^{\text{B}},\;M_{\text{C}}^{\text{B}}$	Number of activated and cycling M cells conjugated to APCs	
$M_{\text{T}}^{\text{B}}$	Total number of conjugated M cells: $M_{\text{T}}^{\text{B}}=M_{\text{A}}^{\text{B}}+M_{\text{C}}^{\text{B}}$	
$M_{\text{A}}^{\text{F}},\;M_{\text{C}}^{\text{F}}$	Number of activated and cycling M cells non-conjugated to APCs: $M_{\text{l}}^{\text{F}}=M_{\text{l}}=M_{\text{l}}^{\text{B}},\;\forall l\in \left\{A,\;C\right\}$	
*F*	Total number of APC conjugation sites that remain free in the system	
SigE, SigR, SigM	Number of bound cytokines signaling receptors at the surface of an activated *E, R*, and *M* cells	

**Symbolic labels**	**Definitions**	

*l*	Symbolic label that denotes the possible functional states of the T cells: *l* = *N* resting state, *l* = *A* activated state, and *l* = *C* cycling state	

**Parameters**	**Definitions**	**Values used in simulations**

Γ*_e_*, Γ*_r_*	Input rate of new resting self-reactive E and R cells from the thymus	2.5 × 10^4^ cells/day
$K_{\text{A}}^{\text{E}},K_{\text{A}}^{\text{R}}$	Activation rate for resting E and R cells conjugated to APCs	Ln(2)/2 h, Ln(2)/6 h
$K_{\text{P}}^{\text{E}},\;K_{\text{P}}^{\text{R}},\;K_{\text{P}}^{\text{M}}$	Division rate for cycling E, R, and M cells	Ln(2)/4 h
$K_{\text{S}}^{\text{E}},\;K_{\text{S}}^{\text{R}}$	IL2 signaling-waiting rate for activated E and R cells	Ln(2)/2 h
$K_{\text{S}}^{\text{M}}$	IL2 signaling-waiting rate for activated M cells	Ln(2)/4 h
$K_{\text{d}}^{\text{E}},K_{\text{d}}^{\text{R}},K_{\text{d}}^{\text{M}}$	Death rate for free resting E and R cells, and free activated M cells	Ln(2)/1 week
*A*	Number of total APCs	2 × 10^5^
*s*	Total number of conjugations site per APC	5
$K^{\text{E}},\;K^{\text{R}}$	Equilibrium conjugation constants (*K*_on_/*K*_off_) for E and R cells to the APC conjugation sites	*K*_on_ = 10^−13^ L s^−1^ cell^−1^, K_off_ = 6 × 10^−4^ s^−1^
*K*^M^	Equilibrium conjugation constants (*K*_on_/*K*_off_) for M cells to the APC conjugation sites	*K*_on_ = 10^−13^ L s^−1^ cell^−1^, K_off_ = 6 × 10^−3^ s^−1^
α_E_, α_R_	Fraction of activated E and R cells reverting to the resting state in the absence of cytokine related signal	0.95
*h*	Hill coefficient at the sigmoid response curve	4
*S*_E_, *S*_R_, *S*_M_	Sensitivities thresholds for E, R, and M cells to cytokines signal	500

**Table 3:** **Variables and parameters related to dynamics of IL2, IL2R, and mAb complexes formation**.

Variables	Definitions	
$C_{\text{j}}^{{\text{E}}_{\text{l}}},\;C_{\text{j}}^{{\text{R}}_{\text{l}}},\;C_{\text{j}}^{{\text{M}}_{\text{l}}}$	Number of IL2 molecules bound to *j* chain of IL2R, at the surface of the indicated T cell type	
${\text{Cm}}_{\text{j}}^{{\text{E}}_{\text{l}}},\;{\text{Cm}}_{\text{j}}^{{\text{R}}_{\text{l}}},\;{\text{Cm}}_{\text{j}}^{{\text{M}}_{\text{l}}}$	Number of IL2m molecules bound to *j* chain of IL2R, at the surface of the indicated T cell type	
${\text{CAb}}_{\text{j}}^{{\text{E}}_{\text{l}}},\;{\text{CAb}}_{\text{j}}^{{\text{R}}_{\text{l}}},\;{\text{CAb}}_{\text{j}}^{{\text{M}}_{\text{l}}}$	Number of IL2/mAb complexes bound to the *j* chain of IL2R, at the surface of the indicated T cell type	
$T^{{\text{E}}_{\text{l}}},T^{{\text{R}}_{\text{l}}},T^{{\text{M}}_{\text{l}}}$	Number of IL2 molecules bound to high affinity IL2R (alpha + beta), at the surface of the indicated T cell type	
${\text{Tm}}^{{\text{E}}_{\text{l}}},\;{\text{Tm}}^{{\text{R}}_{\text{l}}},\;{\text{Tm}}^{{\text{M}}_{\text{l}}}$	Number of IL2m molecules bound to high affinity IL2R (alpha + beta), at the surface of the indicated T cell type	

**Intermediate variables**	**Definitions**	

$P_{\text{j}}^{{\text{E}}_{\text{l}}},\;P_{\text{j}}^{{\text{R}}_{\text{l}}},\;P_{\text{j}}^{{\text{M}}_{\text{l}}}$	Number of IL2R of *j* chain free (not bound to IL2), at the surface of the indicated T cell type	
SigE, SigR, SigM	Number of cytokines signaling receptors at the surface of an activated *E, R*, and *M* cells	

**Parameters**	**Definitions**	**Values used in simulations**

$K_{\text{off}}^{\text{j}},\;K_{\text{on}}^{\text{j}}$	Dissociation and association constant of IL2 to the *j* chain of the IL2R	$K_{\text{off}}^{\alpha }=0.6\;{\text{s}}^{-1}$, $K_{\text{on}}^{\alpha }=10^{7}M^{-1}{\text{s}}^{-1}$, $K_{off}^{\beta }=3\times 10^{-3}{\text{s}}^{-1}$, $K_{\text{on}}^{\beta }=3.4\times 10^{6}M^{-1}{\text{s}}^{-1}$
*f*_j_	Parameter that control the properties of different IL2m	10^−3^, 10^3^
*N*_j_	Switch parameter setting if the mAb blocks (=1) or not (=0) the interaction of IL2 with the *j* chain of the IL2R	0, 1
${\text{ila}}_{E_{A}},{\text{ila}}_{M_{A}}$	Number of cytokine signaling receptors, at the surface of an activated *E* and *M* cells, bounds to an alternative cytokine (not IL2)	10^8^, 10^7^
${\text{Ra}}^{{\text{E}}_{\text{l}}},\;{\text{Rb}}^{{\text{E}}_{\text{l}}}$	Total number of alpha and beta chains of IL2R per E cells in the state *l*	${\text{Ra}}^{E_{N}}=10$, ${\text{Ra}}^{E_{A}}=10^{4}$, ${\text{Ra}}^{E_{C}}=10^{3}$, ${\text{Rb}}^{E_{l}}=10^{3}$
${\text{Ra}}^{{\text{R}}_{\text{l}}},\;{\text{Rb}}^{{\text{R}}_{\text{l}}}$	Total number of alpha and beta chains of IL2R per R cells in the state *l*	${\text{Ra}}^{{\text{R}}_{\text{N}}}=10^{4},\;{\text{Ra}}^{{\text{R}}_{\text{A}}}\text{=1}{\text{0}}^{\text{5}},\;{\text{Ra}}^{{\text{R}}_{\text{C}}}\text{=1}{\text{0}}^{\text{4}},\;{\text{Rb}}^{{\text{R}}_{\text{l}}}\text{=1}{\text{0}}^{\text{3}}$
${\text{Ra}}^{{\text{M}}_{\text{l}}},\;{\text{Rb}}^{{\text{M}}_{\text{l}}}$	Total number of alpha and beta chains of IL2R per M cells in the state *l*	${\text{Ra}}^{{\text{M}}_{\text{l}}}\text{=10,}\;{\text{Rb}}^{{\text{M}}_{\text{l}}}\text{=1}{\text{0}}^{\text{4}}$
$K_{\text{on}}^{\alpha \beta },\;K_{\text{off}}^{\alpha \beta }$	Association and dissociation rates for the interaction of free beta chain to preformed IL2/alpha chain complexes, at the T cell membrane	$K_{\text{on}}^{\alpha \beta }=2.2\;\times \text{1}{\text{0}}^{-3}{\text{s}}^{-1}$, $K_{\text{off}}^{\alpha \beta }=3\times 1{\text{0}}^{-3}{\text{s}}^{-1}$
$K_{\text{on}}^{\beta \alpha },\;K_{\text{off}}^{\beta \alpha }$	Association and dissociation rates for the interaction of free alpha chain to preformed IL2/beta chain complexes, at the T cell membrane	$K_{\text{on}}^{\beta \alpha }=0.6\times 10^{-2}{\text{s}}^{-1}$, $K_{\text{off}}^{\beta \alpha }=0.6\;{\text{s}}^{-1}$
*K*_in_	Internalization (degradation) rate of signaling IL2/IL2R complex by T cells	*K*_in_ = 0.04 min^−1^

The simulations of the model dynamics was implemented using the program Mathematica v.4.0.

## Results and Discussion

### Basic model and simulations setup

The model is setup to study the basic homeostasis of the immune system of a mouse (27). Therefore the APCs in the model are interpreted as those APCs, which present self-antigens to T cells in the absence of infections. In consequence, the CD4^+^ T cells in the model are taken to represent the populations of auto-reactive E and R cells, which significantly recognizes the existent self-antigens and thus interact with the available APCs.

Two main problems are then studied in the model simulations. (a) The basic dynamics states of the system in the absence of treatments; and (b) The effect of perturbations which represent specific IL2 modulation treatments on the stability of these dynamics states.

### Tolerance and immunity as the basic model steady states (in the absence of treatment)

**Figure 4:**
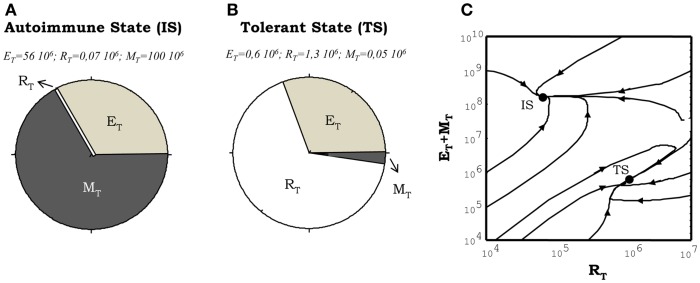
**Illustration of the steady states obtained from numerical simulations of the model**. **(A,B)** shows the proportion of the total T cell number corresponding to helper (E), regulatory (R), and memory (M) T cells. The situation showed in **(A)**, corresponds to the autoimmune steady state (IS) where the memory and helper T cells dominate the system. The situation depicted in **(B)**, correspond to the tolerant steady state (TS) where the regulatory T cells dominate the dynamics. The graph in **(C)** illustrates how these two types of steady state of the system co-exist in the same region of parameter values (the region of bistability). It is shown how different initial conditions, changing just the proportion of E, R, and M cells at time *t* = 0, leads to trajectories taking the system either in the tolerant (TS) or the autoimmune (IS) steady state.

The model has two stable steady states which can be interpreted as natural tolerance and autoimmunity in the system. The steady state, which is interpreted as an autoimmune state (Figure 4A), is one where auto-reactive helper cells are significantly expanded while the auto-reactive Regulatory T cells are outcompeted from their cognate APCs. This steady state is also characterized by the existence of high levels of free IL2 and some subsequence expansion of the memory CD8^+^ T cells population (M cells) in the lymph nodes. The steady state, which is interpreted as natural tolerance in the model, is one where the auto-reactive E and R cells co-exist in a dynamic equilibrium (Figure 4B). In this steady state the expansion of the auto-reactive helper cells is actively controlled by their interaction with the auto-reactive Regulatory T cells, the amount of free IL2 remains very low and the size of M cell population remains close to its basal homeostatic level.

A key dynamical property of the model is the existence of a parameter regime where these steady states of tolerance and autoimmunity can co-exist. This is a regime of bistable behavior (Figure 4C), where the model could evolve dynamically into either to the autoimmune or the tolerant steady state, but depending on the initial conditions used to seed the simulation without any change of parameter values (i.e., changing the initial proportion of auto-reactive E to R cells). The model is set to operate inside this bistable parameter regime. Thus in equilibrium, in the absence of treatments, the system will be either in the tolerant or the autoimmune steady state referred above. Such parameter choice is required to explain properly with the model the results of adoptive transfer experiments in mice, where transferring different proportions of CD4^+^CD25^−^ (helper) and CD4^+^CD25^+^ (regulatory) T cells into immune deficient mice (those lacking T cells, Rag^−/−^ or nu^−/−^), they either reconstitute a normal (tolerant to self-antigens) immune system or develop an autoimmune disease mediated by the uncontrolled expansion of the transferred auto-reactive CD4^+^ T cells (28).

Moreover, it is important to note that the model reviewed here is an extension of the cross-regulation model of immunity, which studies the interaction of helper and regulatory CD4^+^ T cells in the lymph node of the normal mice (33). Interestingly, despite of substantial increase on the number of variable and parameters, the new model conserves the three main dynamical properties of the original one reviewed in Ref. (34). In Ref. (28), three parameter conditions were presented as necessary in the extended model to behave as the original model and therefore to explain the same phenomenology. These conditions are:

(1) Regulatory T cells have to be more efficient using IL-2 at low concentrations than helper and memory T cells.
(2) The existence of a cytokine alternative to IL-2 that promote helper T cell proliferation and survival.
(3) The helper cells must become activated and proliferate more rapidly than Regulatory T cells in conditions of IL-2 excess.

A detailed discussion of the validity of these constrains, from an experimental point of view, is provided in Ref. (28).

### Response to treatments that modulate IL2 concentration

In following sections, the effects of different treatments, which aim to modulate IL2 activity, are studied. Treatments simulate a continuous infusion for a defined period of time of the involved molecules (IL2, IL2m, and/or anti-IL2 antibody). Two parameters control their application: the “dose,” which set up the total amount per day of IL2, IL2m, and/or anti-IL2 antibody infused; and the “treatment duration,” which set the time period of sustained infusion. Treatments are always applied in a system which is initially set to a dynamic equilibrium (i.e., either into the tolerant or the autoimmune steady state). We systematically study, whether a given treatment induces a significant change in the initial proportion of Regulatory (R) versus Helper (E + M) T cells, both transiently or permanently. We interpret that a treatment promotes immunity when it induces a transition from the tolerant steady state (dominated by R cells) to the autoimmune steady state (dominated by E cells). We interpret that a treatment promotes tolerance when it induces a transition from the autoimmune state to the tolerant steady state.

#### Simulating the injection of IL2

**Figure 5:**
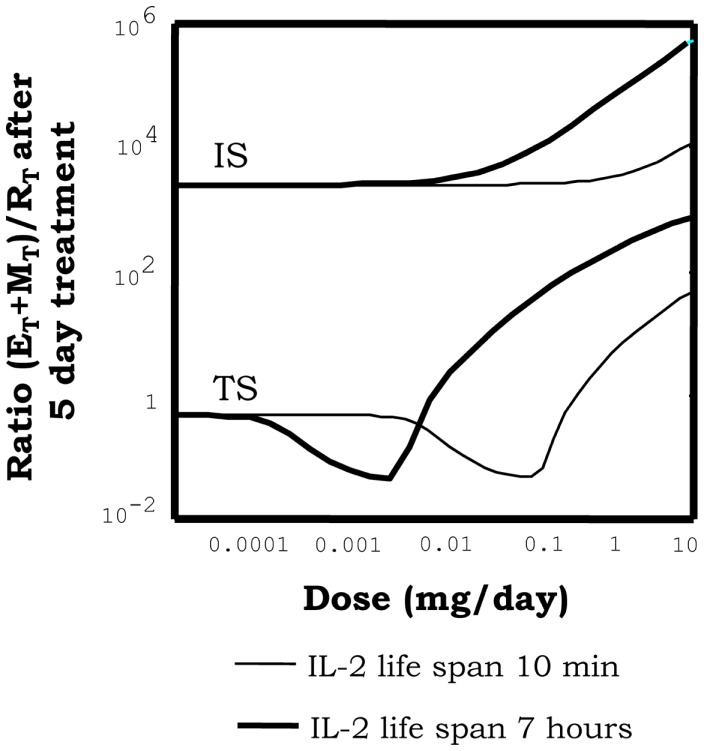
**Effect of injections of IL2, on the proportion of helper + memory T cells versus regulatory T cells [ratio (E + M)/R], in a system initialized either in tolerant (TS) or the autoimmune (IS) steady state**. The graph shows the ratio (E + M)/R attained in the system right after 5 days of continuous injections of the indicated dose (*x* axis of the graph) of an IL2 with either 10 min (thin curves) or 7 h (thick curves) life span in solution. It can be seen how when the simulations start with a system at the TS, the ratio (E + M)/R reduce its values for intermediate dose of the treatment. This is a direct consequence of a preferential expansion of the R cells in the system. However, if the dose is further increased then the ratio (E + M)/R is significantly increased. This is a direct consequence of the expansion of helper and memory T cells, by the treatment application. When the treatment start on a system at the IS, then increasing the dose always leads to an increase of the ratio (E + M)/R. This is, it further increments the number of E and R cells in the system. Interestingly increasing the life span of the injected IL2 moves to lower values the dose ranges where treatment is effective, but does not change the qualitative pattern of response observed.

Simulations of IL2 injections show that, when this treatment is applied to a system initialized into the autoimmune steady state, it is unable to take the system into the tolerant steady state, irrespectively of the dose and treatment duration chosen. Moreover, it further promotes the expansion of the auto-reactive E cells and the M cells (Figure 5) reinforcing transiently the ongoing autoimmune response. However, when this treatment is applied to a system initialized in the tolerant steady state it reinforces the preexistent tolerance, by further expanding the regulatory populations (Figure 5). Interestingly, increasing the IL2 dose applied to a preexistent tolerant state could induce immunity by expanding the M cells; although, this effect is obtained for significantly high (unrealistic) values of the IL2 dose.

Thus overall in the model, IL2 injections appear to reinforce the preexistent steady state, this is expanding transiently either the R or the E cells respectively for a preexistent tolerant or autoimmune situation. A closer look to the model behavior qualitatively explains these results. Briefly: in a preexistent autoimmune steady state there is an excess of IL2 in the lymph node, thus is not lack of IL2 what limits regulatory T cell expansion, is their competition with auto-reactive E cells for the cognate APCs. In consequence injecting IL2 would never reestablish tolerance. In a preexistent tolerant steady state, there is a small amount of IL2 in the lymph node, which is almost exclusively used by the regulatory T cells, limiting their expansion. The helper T cells do not expand as result of the direct suppression of their activation exerted by the R cells. In this situation the injection of IL2, naturally leads to the enhanced expansion of R cells reinforcing the suppression over the E cells. Only when the IL2 concentration is extremely high at the lymph nodes it triggers a significant expansion of the Memory T cells, signaling through the intermediate affinity IL2 receptor beta-gamma. The excessive expansion of the M cells in the system affects the suppressive interaction between E and R cells at the APCs, since these cells, although much weakly, also interact with and compete for the available APCs.

Interestingly the latter model predictions are indeed compatible with existent experimental observations and further provide a guideline for its future practical application. On the one hand, the reinforcement of ongoing immune reactions by IL2 injections, predicted by the model, explains classical observations on *in vivo* animal models, where IL2 have been shown to potentiate immune reactions to viral infection (35) and to well-adjuvated vaccines (1–3). In these systems the immune response induced to the involved foreign antigens, which are most probably loosely or just not controlled by regulatory T cell activity, is further promoted by the injected IL2. Furthermore, the observed enhancement of immunity, in these experimental systems, might not relay just on the model predicted expansion of helper CD4^+^ T cells. It might also involve important direct effects of IL2 on memory CD8^+^ T cell and/or NK cells, which are known to be relevant in many of these particular systems. In any case the model here, will further predict that optimal application of IL2 for the purpose of enhancing immunity, will be obtained when providing IL2 after the immune reaction have already started and never before, because some reminiscent of immune regulation might still exist and could be potentiated by the added IL2.

On the other hand, the capacity of IL2 addition to reinforce natural tolerance mediated by regulatory T cells, predicted by the model, explains as well several experimental observations. Particularly, it explains clinical data stating that regulatory T cells populations are significantly expanded, both in cancer (9, 36) and HIV (37) patients, treated with IL2. Such effect might be related to the poor efficacy observed in these clinical applications of IL2. Particularly, in the case of cancer, less than 20% of the treated patients show some anti-tumor effect, perhaps, according to the model here, because just an small fraction of the patients, happen to have a naturally preexistent immune response against tumor antigens, which could be further enhanced by the injected IL2. In the case HIV patients, IL2 based therapy have led to the recovery of CD4^+^ T cells counts, but the patients do not seem to recover their capacity to fight general infections, perhaps, according to the model here, because this treatment is just reinforcing tolerance mediated by regulatory T cell activity.

Furthermore, this second model prediction also explains many results in pre-clinical animal models. It explains, for instance, that IL2 injections can prevents allograft rejection (10); or attenuate the induction of Experimental Autoimmune Encephalomyelitis (EAE) (10); or fully prevent the development of diabetes in the NOD mice (11). Interestingly, in the EAE and allograft reaction models the latter effects are observed for scheme of IL2 applications where this cytokine is injected in the system before implanting the allogeneic tissue or before inducing the EAE. This is before the immune/autoimmune reaction has been expanded; i.e., when there is a preexistent natural tolerance, mediated by regulatory T cells, which could be reinforced by the applied treatment. However, in the NOD mice model, recent data (12) have shown that IL2 treatment at the onset of diabetes could revert disease development. Interestingly, in this “therapeutically relevant scenario” treatment efficacy is much lower than in the preventive settings. Only 40–60% of the NOD mice appear to be cured, while 100% of the NOD mice are diabetes free when treating in the preventive settings. Whether or not at the onset of NOD mice diabetes the balance between regulatory T cells and helpers T cells have been fully disrupted in favor of immunity, just as considered in our model simulations of an autoimmune disease therapeutic scenario, is a matter of discussion. Actually Grinberg-Bleyer et al. have shown that at the onset of NOD diabetes a significant amount of regulatory T cells can still be found in the pancreas and its draining lymph node. Unfortunately in the NOD mice, the acute nature of diabetes development (with a full irreversible destruction beta islet) invalidates any displacement of the treatment application toward a more advanced stage of the disease, to better compare with our model predictions.

#### Simulating the injections of different anti-IL2 mAbs

Anti-IL2 antibodies are molecules that form complexes with the IL2, blocking or not its binding to the different chains of the IL2 receptor at the T cell surface and therefore interfering with the associated signaling process. Three classes of antibodies are systematically explored in our simulations following its documented existence in the literature (20, 38): (1) The anti-IL2 mAbs, which bind and thus block the site in the IL2 surface implicated on the interaction with the alpha chain of the IL2 receptor (referred here as the face alpha mAbs); (2) The anti-IL2 mAbs, which bind and thus block the site in the IL2 surface implicated on the interaction with the beta chain of the IL2 receptor (referred here as face beta mAbs); and (3) the anti-IL2, which block the binding of IL2 to all chains of the IL2 receptor (referred here as a fully blocking mAbs).

**Figure 6:**
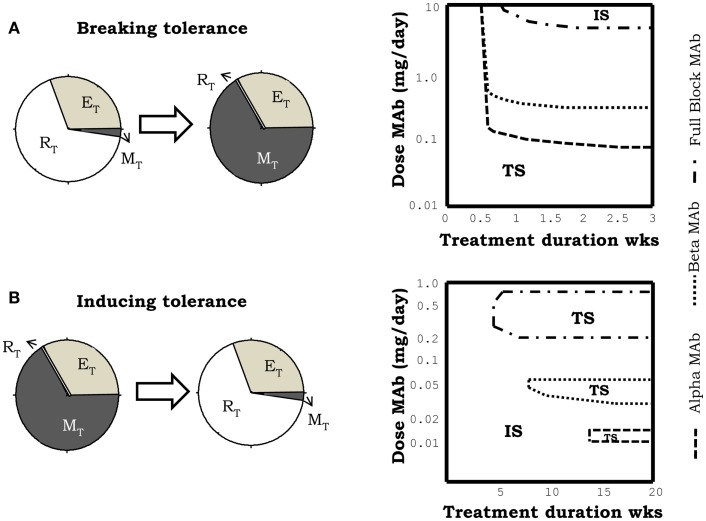
**Effect of the simulation of treatments of IL2 depletion, using different anti-IL2 antibodies**. mAbs in the simulation, can block the interaction of IL2 with the alpha (face alpha mAb), or with the beta (face beta mAb) or with both (fully blocking mAb) chains of the IL2R. The graphs in **(A)** corresponds to the case in which the treatment induces a breakdown of the preexistent tolerant steady state, i.e., a transition to the autoimmune steady state. The graphs in **(B)** corresponds to the case in which treatment induces tolerance, taking into the tolerant steady state, a system initially set in the autoimmune steady state. Breakdown of a preexistent tolerant state requires a minimal effective dose of mAb and treatment duration [graph in the right side of **(A)**]. In this scenario, face alpha mAbs are more efficient than face beta or fully blocking mAb. This means that the latter need higher doses of mAb to achieve a similar effect. Induction of tolerance requires minimum treatment duration with a mAb dose inside an intermediate window of values [graph in the right side of **(B)**]. This effect is obtained when face alpha, face beta, or fully blocking mAbs are used.

The injection of monoclonal antibodies anti-IL2, in the model simulations, when applied to a previously tolerant system could induce a breakdown of tolerance (Figure 6A), with the consequent transition of the system to the autoimmune steady state. Such effect can be obtained with the three classes of anti-IL2 mAbs studied, but it requires a minimal effective dose of the anti-IL2 mAb and treatment duration (Figure 6A) which varies significantly with the type of mAbs used. Face alpha mAbs are significantly more efficient than fully blocking or face beta mAbs (Figure 6A) in this simulation. Moreover, for the three classes of mAbs studied the higher the affinity for the IL2 the higher their efficacy in these simulation (27).

The effect of treatment with anti-IL2 mAbs in a system with a preexistent autoimmune reaction is also quite significant. In this case, the treatment is capable of resetting the system into the tolerant steady state (i.e., inducing tolerance) (Figure 6B). This effect occur under quite restrictive treatment conditions: there is a minimal treatment duration required and the dose of the anti-IL2 mAbs used has to be set inside some particular intermediate range of values (Figure 6B). The tolerogenic effect of the anti-IL2 mAbs is obtained with all type of mAbs (Figure 6B). Differences in the mAbs affinity and mAbs class strongly change the dose range where this effect is observed^2^.

Overall the simulations of IL2 depletion treatments using anti-IL2 antibodies predict that this type of therapy is able to break a preexistent tolerant state, inducing an autoimmune response, or to render tolerant a preexistent autoimmune system. A closer look to the model behavior qualitatively explains these results as follows. The injected mAbs appear to sequester the IL2, limiting its availability to provide signal to the T cells. When the treatment is applied into an initially tolerant steady state, the initial effective concentration of IL2 is low and it is further reduced to insignificant levels, where this cytokine is incapable to signal neither to E, R, or M cells. Therefore, if the treatment is sustained long enough, the number of R cells fall down to a minimum determined by the size of the thymic output, because the proliferation and survival of R cells is strictly dependent on IL2. But the number of E cells, on the other hand, set back to a value determined by the availability of the homeostatic cytokine of ILα, which they could use as alternative to IL2 signal. Therefore once the injected mAbs are cleared, the auto-reactive E cells could have some initial advantage in respect to the R cells, leading the T cell expansion, which drive the system into the autoimmune steady state. However, when the treatment is applied to an initially autoimmune system, the effective concentration of IL2 is quite large and it is reduced by the presence of the antibody. The efficacy of the mAbs to affect IL2 signaling in the different T cell population is strongly dependent on its affinity for the IL2 and the side of the IL2 recognized. For a very high antibody dose, the effective IL2 concentration falls to negligible values, which as before are unable to signal neither to E, R, or M cells. Thus the size of the auto-reactive E cell population is reduced to the value set by the availability of ILα and the number of R cell remains low in a value determined by the size of thymic output. When the injected antibody is cleared the system could return back to the autoimmune equilibrium. However, for some intermediate doses of the antibody, the effective IL2 concentration is reduced to values where it is unable to signal on the E and M cells, but it is still significant for the R cells, which are more sensitive due to their higher expression of the alpha chain of the IL2 receptor. Therefore, for these mAbs doses the E cell population is reduced to the minimal size, which can be sustained by the available ILα. But the R cells are stimulated to grow forcing the system to switch into the tolerant steady state.

The model prediction of a higher efficacy of treatments with face alpha mAbs, to break a preexistent tolerant steady state, relates to the impact of this type of mAbs on the dynamics of the M cells. Face alpha mAbs bind the available IL2 forming immune-complexes that can still signal through the intermediate affinity IL2 receptor (beta + gamma chain). This form of the receptor is prevalent in the M cells, thus face alpha mAbs partially redirect IL2 signaling into the M cells expanding this population. The growth of the M cells interferes with the dynamics of CD4^+^ T cells, i.e., M cells consume the available IL2 and reduce the capacity of CD4^+^ T cells to interact with the APCs. The combination of the latter effects explains the advantage of the face alpha mAb to break a preexistent tolerant steady state. On the other hand, the differences observed between fully blocking and face beta mAbs in the model simulations (compare dose dependencies in Figure 6), must rely on the fact that face beta mAbs do not block the interaction with the alpha chain of the IL2 receptor, conditioning the attachment of the immune-complexes formed to cells that express this molecule at the cells surface. These interactions significantly alter the bio-distribution of both the IL2 and the antibody.

Interestingly, the latter model predictions are indeed compatible with existent experimental observations. On the one hand, the predicted capacity of treatments blocking IL2 activity to promote autoimmunity/immunity, explains observations where monoclonal antibodies against IL2 have been shown to promote effective immune responses to tumors (16) and to induce autoimmune disease in naïve mice (13). In both cases, the model explains the observed effects as being associated to the treatment capacity to weaken regulatory cell activity, just as argued by their original authors. It must be also noted that in these reports the S4B6 anti-IL2 mAbs was used, a mAb which has been recently proven to block only the interaction of IL2 with the alpha chain of the IL2 receptor (38).

**Figure 7:**
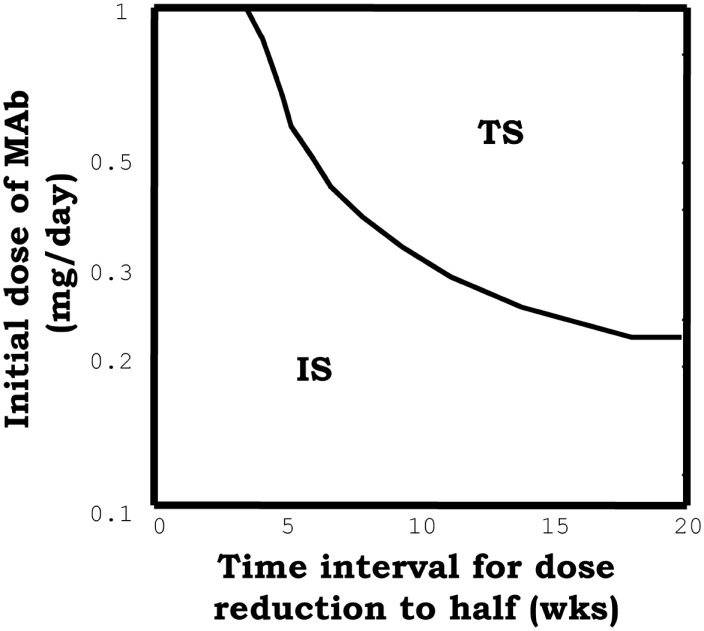
**The graph summarizes the results of simulations of a model system set initially to the IS and then perturbed with, an initial dose of the face beta mAb, which is periodically reduced to the half at the indicated time (*x* axis)**. The curve indicates the minimal value of the initial mAb dose required to induce tolerance (taking the system into the tolerant steady state) with the applied treatment.

On the other hand, the model predicted capacity of IL2 blocking therapies to reestablish tolerance in the context of ongoing immune/autoimmune reactions, is not documented in the literature. This model prediction is very interesting from the practical perspectives for the treatment of autoimmune diseases. However, the fact that the predicted treatment effect just occurs for a particular intermediate range of antibody doses, applied during a relatively long period of time, makes difficult the practical implementation of the treatment. To overcome the latter problem we suggested, based on model simulations, an alternative/simpler strategy to capitalize this therapeutic effect. A large initial dose of the mAb could be used, reducing it periodically with a fixed rate. With this alternative strategy the model predict a much simpler dose dependency (see Figure 7) of treatment efficacy, i.e., the applied initial dose used must be large enough (as to induce significant initial immunosuppression), and the reduction rate used should be sufficiently slow.

#### Simulating the injection of IL2/mAb immune-complexes

Immune-complexes of IL2 plus anti-IL2 mAbs (in a 1:2 mAb:IL2 molar proportion), has been recently highlighted as a novel therapeutic strategy (18, 20, 39) which could significantly potentiate the activity of the IL2 *in vivo*. Intuitively it should be expected that the therapy with immune-complexes share properties with the therapies based on its basic components, but their comparative efficacy shall depend on the class and the affinity of the mAbs used.

**Figure 8:**
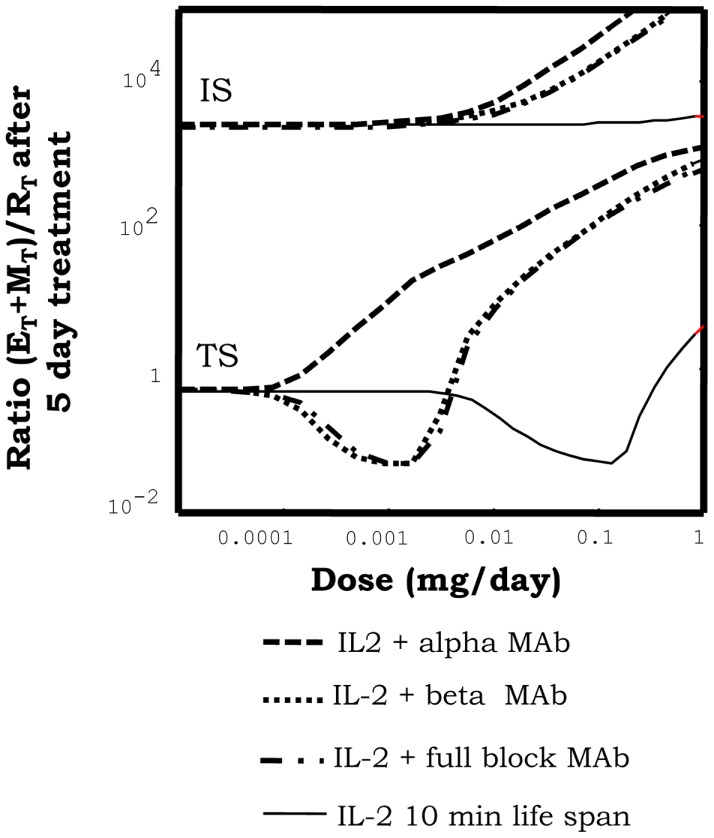
**Effect of injections for five days of the indicated doses of immune-complexes of IL2 plus antibodies anti-IL2, on the ratio of helper + memory T cells versus regulatory T cells [ratio (E + M)/R], in a system initialized either in tolerant (TS) or the autoimmune (IS) steady state**. Different immune-complexes differ on the class of mAb used to form it (face alpha, face beta or fully blocking mAbs). immune-complexes are always formed with a 1:2 molar ratio of mAbs:IL2 and the dose applied is reported in terms of the mass of IL2 injected. If the simulations start with a system at the TS, immune-complexes formed with face beta or fully blocking mAbs reduce the ratio (E + M)/R for some intermediate dose values and then increases it for higher dose values. This is a pattern of response, qualitatively similar to that obtained with IL2 injection, but significantly displaced to the range of lower doses of IL2. If face alpha mAbs are used to form the complex the pattern of response obtained is qualitatively different. The ratio (E + M)/R always increase (favoring the expansion of E and M cells) and the larger the dose applied the larger the increment. If the simulations start with a system at the IS, all the possible immune-complexes behave qualitatively like the IL2 alone, they promote in dose-dependent way a further expansion of E and R cells, increasing the ratio (E + M)/R.

**Figure 9:**
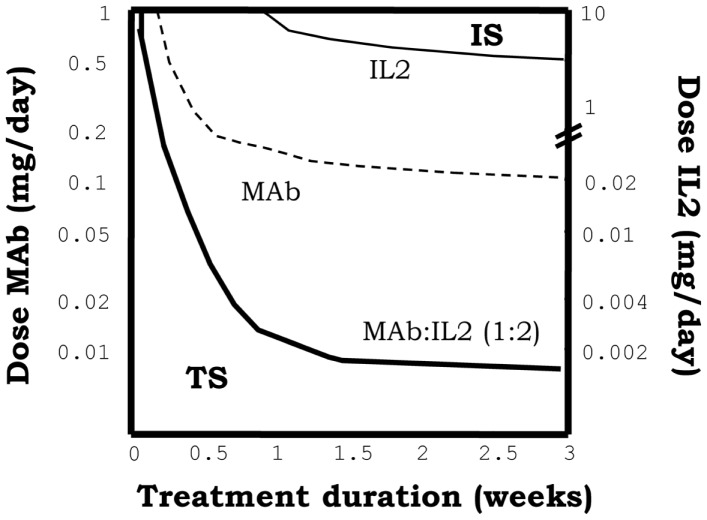
**The graph shows the minimal effective dose of mAb (left *y* axis) or IL2 (right *y* axis) versus treatment duration, required to induce the transition to the IS in a system initialized in the TS, for the treatment with immune-complexes formed with face alpha mAbs in the optimal molar proportion 1:2 (mAb:IL2)**. For direct comparison the equivalent curves obtained for treatments with the same mAbs alone or the IL2 alone are also depicted. It can be seen that the injection of this class of immune complex is more efficient than the injection of the mAb or the IL2 alone to breakdown tolerance in an initial tolerant system, i.e., it requires less dose of either the mAbs or IL2 as compared to the independent treatments.

In our simulations, immune-complexes can either reinforce or weaken a preexistent tolerant steady state depending on the class of mAb used on its formulation. Figure 5, shows how the injection of immune-complexes significantly changes the number of E, R, and M cells in the system initially set to the tolerant steady state. Immune-complexes formed with beta face or fully blocking mAbs induce a quite significant transient expansion of Regulatory T cells (reinforcing the tolerant state). This transient expansion of the Regulatory T cells is significantly larger than the one induced by an equivalent treatment with IL2 alone and it is maximal for mAbs with some intermediate affinity for the IL2 (27). However, immune-complexes formed with face alpha mAbs have a quite different effect in these simulations (Figure 8). They could also expand the R cells, but they expand much more in comparison the M cells in the lymph node. The capacity of this immune complex to expand M cells became larger as their affinity for the IL2 is increased (27). Interestingly for the three classes of immune-complexes a sufficiently high dose of the latter treatment could induce a breakdown of tolerance. But only immune-complexes based on face alpha mAbs perform better in this task than the therapy based on the anti-IL2 mAb or the IL2 alone (Figure 9).

When applied to initially autoimmune steady states, all immune-complexes fail to reestablish tolerance steady state. As the injection of IL2 the immune-complexes further reinforce a preexistent autoimmune steady state, expanding the helper and memory T cells (Figure 8).

Summarizing the results above shows that immune complex can sometimes synergistically potentiate the effects of IL2 and mAbs. Complexes based on face alpha mAbs do promote immunity primarily by expanding the M cells, and leading ultimately to a quite efficient breakdown of a preexistent tolerant steady state. Complexes based on face beta mAbs, can efficiently reinforce tolerance expanding significantly the R cells preexistent in the tolerant steady state. Face alpha mAbs for immune-complexes are better with the highest possible affinity, but face beta mAbs could be better with some intermediate affinity values.

Qualitatively the effects of immune-complexes can be explained based on two main dynamical properties in the model: (A) In the immune complex the IL2 is protected from degradation. While bind to the mAbs the IL2 has a life span of 3 days (like the mAbs), which is significantly larger than the life span of 10 min reported for free IL2. (B) Immune-complexes block different sites in the surface of IL2 conditioning its preferential interaction with different cell populations, accordingly to their differential expression of the IL2 receptor chains. Face alpha mAbs, form immune-complexes that bind and signal through the beta + gamma pair of IL2 receptors. Thus, since beta chain is over-expressed by the M cells, this complex preferentially redirect the IL2 signal to these cells. Following this analysis one could easily explain why this type of immune complex has a maximal efficiency when the affinity of the face alpha mAbs used is high. With high affinity mAbs, the IL2 is more protected from degradation, and the signaling is maximally redirected to the M cells. Face beta mAbs form immune-complexes unable to signal in any class of IL2 receptor. Thus to mediate any biological activity this type of complex has to partially dissociate, working as a controlled source of free IL2. If the affinity of the face beta mAbs in the complex is too high then the IL2 is never released and the immune-complexes have no effect at all. If the affinity of the face beta mAbs is too low, then injecting the complex is like injecting IL2 alone. However, if the affinity of the face beta mAbs in the complex is larger than the affinity of the dimeric IL2 receptor (beta + gamma chain), but lower than the affinity of the trimeric IL2 receptor (alpha + beta + gamma chain), the IL2 in the complex is easily release to provide signal through the high affinity trimeric IL2 receptor, but not through the intermediate affinity dimeric IL2 receptor. In this way the face beta based immune-complexes provided a preferential signaling to the regulatory cells, which overexpress the alpha chain of the IL2 receptor.

Interestingly the model results explain available pre-clinical data on the use of immune-complexes of IL2-anti-IL2 mAbs. Our observations that immune-complexes formed with face alpha or face beta mAbs expand different cell populations when injected *in vivo* into a normal (tolerant) mouse are fully compatible with the results reported in Ref. (18, 20, 39). In these experiments, the S4B6 mAb (a face alpha mAbs) is shown to form immune-complexes that strongly expands CD8^+^CD44^+^ T cells and to a lesser extent the R cells (20). This face alpha immune complex has been also used to increment the immune response induced with a vaccine (17), showing a significantly higher efficiency than IL2 alone. Moreover, the group of Jonathan Sprent have shown that JES6-1 (originally described as a face beta mAbs (20), although it has been recently observed that it also blocks the interaction with CD25, behaving more like a fully blocking mAbs) induce a larger expansion of Tregs (CD4^+^CD25^+^Foxp3^+^ T cells) than the injection of IL2 alone in the same experimental setting (20). Remarkably, this latter type of immune complex has been shown to reinforce a preexistent tolerance state, preventing graft rejection or autoimmune disease induction in mice (18). But it showed no effect when applied in a therapeutic setting, this is just after the onset of the autoimmune disease or the initiation of skin graft rejection process (18).

The simulations, however, propose some interesting guidelines to improve the therapeutic effect of immune-complexes. They predict that in the case of complexes using face alpha mAbs, the best strategy is to use mAbs with the higher affinity available. But in the case of immune-complexes formed with face Beta or fully blocking mAbs, the use of intermediate affinity mAbs is recommended. Other important prediction of our model simulations is that treatment with immune-complexes based on face beta or fully blocking mAbs are useful to reinforce a preexistent tolerant state preventing the induction of autoimmunity, but it would be quite inefficient to therapeutically treat an already established autoimmune disorder. For the later task, the best strategy would be to use the anti-IL2 mAbs alone following the strategies described in Section “Simulating the Injections of Different Anti-IL2 mAbs.”

#### Simulating the injection of IL2 mutants

Several mutant variants of IL2 have been designed aiming to improve the therapeutic efficacy of wild-type IL2 in cancer therapy. Most strategies, so far explored, involve the development of IL-2 variants with an either reduced or increased binding affinity for the alpha or the beta chain of the IL2R. In this section three particular classes of mutants are simulated: (a) IL2 Mutant with a reduced conjugation affinity for the alpha chain of the IL-2R as the one described in Ref. (40) (referred here as No-alpha mutants); (b) IL2 Mutant with an increased conjugation affinity for the alpha chain of IL-2R as the one described in Ref. (41) (referred here as Alpha-Plus mutants); (c) IL2 Mutant with an increased affinity for the beta chain of the IL2R as the one described in Ref. (42) (referred here as Beta-Plus mutant). These three classes of IL2 mutants provide a functional IL2 signal to T cells, since they keep binding beta and gamma unit of the IL2 receptor (i.e., they are IL2 agonists). But they might be expected to alter the natural balance in which wild-type IL2 is consumed by different T cell types, resulting on a significantly different overall dynamics.

**Figure 10:**
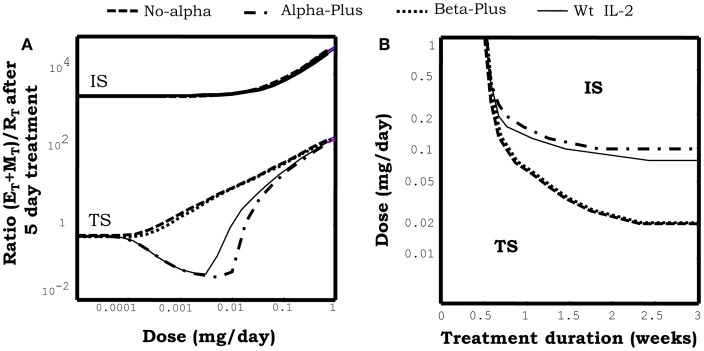
**The graph in (A) shows the effect of injections for 5 days at the indicated dose of different mutant variants of IL2 on the ratio of helper + memory T cells versus regulatory T cells [ratio (E + M)/R], in a system initialized either in the tolerant (TS) or the autoimmune (IS) steady state**. Mutants differ on their capacity to bind to the different chains of the IL2R. Alpha-plus and Beta-plus mutants have higher binding affinity than wild-type IL2 respectively for the alpha chain (*f*_α_ = 1000, *f*_β_ = 1) and the beta chain (*f*_α_ = 1, *f*_β_ = 1000), while No-alpha mutant lack the binding to the alpha chain (*f*_α_ = 0.001, *f*_β_ = 1). All mutant variants were simulated with a life span of 7 h. If the simulations start with a system at the TS, Alpha-plus mutant reduces the ratio (E + M)/R for some intermediate dose values and then increases it for higher dose values. This is a pattern of response, qualitatively similar to that obtained with IL2 injection, although with a slighter wider range of treatment dose with ratio (E + M)/R reduced from its starting value. If No-alpha or Beta-plus mutants are used the pattern of response obtained is qualitatively different. The ratio (E + M)/R always increase (favoring the expansion of E and M cells) and the larger the dose applied the larger the increment. If the simulations start with a system at the IS, all the mutants variants behave like the wild-type IL2, they promote in dose-dependent way a further expansion of E and R cells, increasing the ratio (E + M)/R. The graph in **(B)** shows the minimal effective dose versus the treatment duration, required to induce the transition to the IS in a system initialized in the TS, for the treatment with different variants of IL2 mutants. It can be seen that the injection of No-alpha and Beta-Plus IL2 mutants is more efficient than the injection of wild-type IL2 alone to breakdown tolerance in an initial tolerant system, i.e., it requires less dose to achieve a similar effect.

Figure 10A show the effect of injecting different IL2 variants in a system initially set in the tolerant steady state. As described in Section “Simulating the Injection of IL2,” injection of wild-type IL2 transiently reinforce this preexistent tolerant steady state, preferentially expanding the Regulatory T cells in the system. Alpha-Plus IL2 mutants, exhibit a similar response pattern than wtIL2, but with an even more marked preferential expansion of the regulatory T cells. In contrast, No-Alpha and Beta-Plus mutants show a completely different response pattern than wild-type IL2. This class of mutants expand preferentially the helper T cells (E + M), rather than the regulatory T cells at all injection doses. Moreover injections of the three classes of mutants, as for the wild-type IL2, could lead to a breakdown of tolerance in the system when the dose used is significantly increased. However, the minimal dose required for such effect is significantly lower for the No-Alpha mutants and Beta-Plus mutant Figure 10B, than for wild-type IL2 and Alpha-Plus mutants.

Figure 10A also shows the effect of injecting different classes of IL2 mutants in a model system initially set in the autoimmune steady state. In this case the three classes of mutants behave quite similarly to wild-type IL2, i.e., None of them is able to promote a transition to a tolerant steady states, at any dose and treatment duration. Moreover, they reinforce the preexistent autoimmune steady state, further expanding the Helper and Memory T cells.

Overall the result in this section show that No-Alpha and Beta-Plus IL2 mutants behave quite similarly, being significantly better than wild-type IL2 to promote immunity. While Alpha-Plus mutants could be slightly better that wild-type IL2 to reinforce a preexistent tolerant state, expanding more the regulatory T cells. Qualitatively, the latter results could be easily understood in the model, by taking into account the differential expression of the high affinity/trimeric form (alpha + beta + gamma) and intermediate affinities/dimeric form (beta + gamma) of the IL2 receptors on the different T cell populations. Regulatory T cells, relay on the overexpression of the alpha chain of the IL2R, to have the highest expression of the high affinity form of the IL2R. Memory T cells relay in the overexpression of the beta chain of the IL2 R to have the higest expression level of the intermediate affinity form of the IL2R. The No-Alpha and Beta-Plus mutants have a similar impact in the balance of use of IL2 related signal in the model. In both cases the resulting mutants lack the preferential capacity to signal over Regulatory T cells at low concentration, which is characteristic of the wild-type IL2. Furthermore they will preferentially redirect the signal toward the memory T cells, and strongly promote immunity. As the reverse case the Alpha-Plus mutant, reinforce the capacity of the wild-type IL2 to signal preferentially over the Regulatory T cells, resulting on a better tool to reinforce a preexistent tolerance state.

The results obtained above are compatible with existent experimental data. Both No-alpha (40) and Beta-Plus (42) mutants have been shown to induce a more potent anti-tumoral response than wild-type IL2 in several transplantable tumor models in mice. The dynamic effects predicted *in silico* for these types of IL-2 mutants is qualitatively similar to those described in Sections “Simulating the Injection of IL2/mAb Immune-Complexes” for treatments with immune-complexes of IL-2 and anti-IL-2 mAbs, when face alpha mAb are used. Indeed No-Alpha mutants can be easily conceptualized as an extreme case of such immune-complexes if the affinity of the mAbs for the IL2 tends to infinity. From a quantitative point of view, in the model, IL2 mutants can become as efficient as the immune-complexes, only when its life span is set to be greater than 24 h. If the life span of the mutant is taken to be of 7 h (the one used in Figure 10), which is the one reported for a wtIL2 fused to a constant region of IgG (43), then one might need around 5–10 time more mutant than wtIL2 in the immune-complex to obtain an equivalent effect. However, since immune-complexes work *in vivo* at very low concentrations of IL2, 1–2 micrograms in mice (20), a quite reasonably small amount of the IL2 mutants would be required to induce a similar effect. Thus, these IL2 mutants can be useful tools to promote immunity, for instance to treat tumors or to enhance the response to cancer vaccines. It is important to note that mutants might have some regulatory/developmental advantages as potential drugs in comparison to the immune-complexes, given the fact that they are single molecules.

The predicted capacity of Alpha-Plus mutants to reinforce preexistent tolerant steady state, expanding the regulatory T cells, has never been evaluated. A mutant variant of IL2 with 1000 times’ higher affinity for the IL-2Ra was developed by Rao et al. (41). But only the *in vitro* effect of this mutant on different T cell lines was evaluated. Potentially the Alpha-Plus mutant could be used to treat patients that would receive an organ transplant to reduce the risk of graft rejection. However from a quantitative point of view the mutant efficacy expanding regulatory T cells is predicted only as slightly better than that of wild-type IL2. Moreover, it is quite similar to that obtained with immune-complexes of IL2-anti-IL2 mAbs formed with face beta or fully blocking mAbs (see Simulating the injection of IL2/mAb immune-complexes), when its life span is set to be of around 7 h (the value used in Figure 10). This is when its life span is similar to that reported for a wtIL2 fused to a constant region of IgG (43). Therefore this Alpha-plus mutant or just simply the wild-type IL2 fused to Fc of IgG, could be a reasonable drug to prevent allograft rejection. They might have a similar effect to that reported for immune-complexes in mice, but being much simpler drugs to develop. Indeed a version of IL2 fused to Fc portions of immunoglobulin is already available (43).

## Concluding Remarks

Mathematical modeling of the IL2 and T-cell dynamics, considering the dual role of IL2 in its interaction with regulatory and helper CD4^+^ T cells, is able to explain the complexity observed in the effects of IL2 modulating treatments. In this sense, we show that the model explains a large amount of available clinical and pre-clinical data. Moreover, it predicts optimal strategies for the future application of these treatments:

(A) Mutant variants of IL2, either with reduced affinity for CD25 (the alpha chain of IL2 receptor) or an increased affinity for CD122 (the beta chain of IL2 receptor), and with an increased life span in circulation (for instance fusing them to Fc portion of IgG), are the best strategy to potentiate immunity alone or in combination with vaccines.
(B) Increasing IL2 life span in circulation, either by fusing it with larger proteins or forming complexes with mAbs that block the interaction of IL2 and CD122 (the beta chain of the IL2 receptor), significantly potentiate its capacity to reinforce a preexistent natural tolerance, further expanding the regulatory T cells. This effect might be useful to treat patients that would receive an organ transplant, reducing the risk of graft rejection.
(C) Anti-IL2 antibodies which block the interaction of IL2 with CD122, CD25, or both can be used to treat an ongoing autoimmune disorder, promoting the induction of tolerance. The best schedule for this therapy is to start treatment with a high dose of the mAb (one capable to induce some immune suppression) and then scale the dose down slowly the dose in subsequent applications.

Last, but not least, it is important to highlight that our model has focused on the control that IL2 exerts on T cell cycle progression, impacting both in T cell proliferation and survival. We have neglected some other reported roles of IL2 in T cell differentiation. For instance, IL2 has been reported to increase the suppressive capacity of the Regulatory T cells (12); to condition the differentiation of CD8 T cells into a memory phenotype (44, 45); to induce together with TGFb, the generation of the so called induced Tregs from naïve CD4^+^ T cells (46). We believe these phenomena, although important in some experimental contexts, are not essential to understands the main phenomenology studies in this paper. In future studies, the current model could be extended to include some or all of the above referred interactions of IL2.

Moreover, severe toxicity, i.e., the appearance of the cytokine storm and the vascular leak syndrome, is perhaps the major limitation known today of the practical application of IL2 modulation treatments in clinics. Our model cannot be used to simulate directly the toxic effects of the different IL2 modulation treatments studied. It could only be used to predict strategies that optimize the expected therapeutic efficacy related to the balance between regulatory and effector CD4^+^ T cells. However, a recent report by the group of Boyman (47) has shown that vascular leak syndrome, which leads to severe pulmonary edema, is caused by the direct interaction of IL2 with its high affinity receptor expressed in lung epithelial cells. They demonstrated that treatment with immune-complexes of IL2 + S4B6 mAbs (anti-IL2 mAb which interferes the binding of IL2 to the alpha chain of IL2 receptor), prevents vascular leak syndrome while inducing a potent anti-tumor response. Furthermore, in Carmenate el al. (40), treatment with IL2 mutants with a reduced affinity for CD25 (no-alpha mutant) was shown to be less toxic than treatment with wild-type IL2. These experimental observations support the practical feasibility of some of our model predictions.

## Conflict of Interest Statement

The authors dclare that the research was conducted in the absence of any commercial or financial relationships that could be construed as a potential conflict of interest.

## Footnotes

^1^Note that, although other cytokines are able to stimulate Tregs *in vitro*, several reports in the literature have indicated IL2 as the key cytokine for the proliferation and survival of Treg cells *in vivo*. The group of Freitas (24) have shown that the absence of CD4+ T cells capable of producing IL2, leads to the absence of Treg cells and to the development of autoimmunity. Moreover, mice knockouts of IL2 or IL2 receptor components have been shown to lack the accumulation of Tregs *in vivo*, exhibiting once more an autoimmune phenotype (48, 49). Interestingly in these latter scenarios of autoimmune mice, other cytokines besides IL2 are capable to maintain and expand the auto-reactive helper CD4+ T cells (perhaps IL7, IL15, or IL21).

^2^Treatment using the face alpha mAbs is the best option (it work for lower MAb dose windows, example shown in Figure 6B) when the mAb used has an affinity for IL2 lower than 1010 M−1. But the capacity of this mAb to revert an autoimmune state is completely lost if the mAb affinity for IL2 is assumed to be higher. The face beta mAbs seems to work well for a larger range of mAbs affinities. Its effect is lost only for unrealistically high affinities (larger than 1011 M−1) and it is always better than the one obtained with a fully blocking mAb. In Ref. (27), Figure 7, we presented results considering a mAb affinity higher than 1010 M−1, where the face alpha mAb is not effective in reverting an autoimmune state.

## Appendix A

### Dynamics in the blood compartment

Equations for the dynamics in the blood compartment are the following:

(A1) $$\begin{array}{llll} \frac{d\text{IL}{\text{2}}_{\text{S}}}{dt} & =K_{\text{off}}^{\text{Ab}}\times {\text{IL2}}_{\text{S}}^{\text{Ab}}-K_{\text{on}}^{\text{Ab}}\times \frac{1}{N_{\text{A}}\times V_{\text{S}}}\times \text{IL}{\text{2}}_{\text{S}}\; & & \;\\ & \;\times \text{A}{\text{b}}_{\text{S}}+N_{\text{LN}}\times \left(D_{\text{il}}\frac{\text{IL2}}{\text{fve}\times V_{\text{LN}}}-D_{\text{il}}\frac{\text{IL}{\text{2}}_{\text{S}}}{V_{\text{S}}}\right)\; & & \;\\ & \;-K_{\text{di}}\times \text{IL}{\text{2}}_{\text{S}}+\Gamma_{\text{i}} \end{array}$$

(A2) $$\begin{array}{llll} \frac{d\text{IL2}{\text{m}}_{\text{S}}}{dt} & =N_{\text{LN}}\times \left(D_{\text{il}}\frac{\text{IL2m}}{\text{fve}\times V_{\text{LN}}}-D_{\text{il}}\frac{\text{IL2}{\text{m}}_{S}}{V_{\text{S}}}\right)\; & & \;\\ & \;-K_{\text{di}}\times \text{IL2}{\text{m}}_{\text{S}}+\Gamma_{\text{mi}} \end{array}$$

(A3) $$\begin{array}{llll} \frac{d\text{A}{\text{b}}_{\text{S}}}{dt} & =K_{\text{off}}^{\text{Ab}}\times {\text{IL2}}_{\text{S}}^{\text{Ab}}-K_{\text{on}}^{\text{Ab}}\times \frac{1}{N_{\text{A}}\times V_{\text{S}}}\times \text{IL}{\text{2}}_{S}\; & & \;\\ & \;\times \text{A}{\text{b}}_{\text{S}}+N_{\text{LN}}\times \left(D_{\text{ab}}\frac{\text{Ab}}{\text{fve}\times V_{\text{LN}}}-D_{\text{ab}}\frac{\text{A}{\text{b}}_{\text{S}}}{V_{\text{S}}}\right)\; & & \;\\ & \;-K_{\text{da}}\times \text{A}{\text{b}}_{\text{S}}+\Gamma_{\text{ab}} \end{array}$$

(A4) $$\begin{array}{llll} \frac{d{\text{IL2}}_{\text{S}}^{\text{Ab}}}{dt} & =-K_{\text{off}}^{\text{Ab}}\times {\text{IL2}}_{\text{S}}^{\text{Ab}}+K_{\text{on}}^{\text{Ab}}\times \frac{1}{N_{\text{A}}\times V_{\text{S}}}\times \text{IL}{\text{2}}_{\text{S}}\; & & \;\\ & \;\times \text{A}{\text{b}}_{\text{S}}+N_{\text{LN}}\times \left(D_{\text{ab}}\frac{\text{IL}{\text{2}}^{\text{Ab}}}{\text{fve}\times V_{\text{LN}}}-D_{\text{ab}}\frac{{\text{IL2}}_{\text{S}}^{\text{Ab}}}{V_{\text{S}}}\right)\; & & \;\\ & \;-K_{\text{da}}\times {\text{IL2}}_{\text{S}}^{\text{Ab}} \end{array}$$

Equations A1–A3 model the dynamics of IL2 (IL2_s_), IL2 mutant variants (IL2m_s_), and anti-IL2 antibodies (Ab_s_) number respectively, while the dynamics of the number of immune-complexes IL2 + anti-IL2 antibodies $\left(\text{IL}2_{\text{s}}^{\text{Ab}}\right)$ is modeled by Eq. A4. The variables and parameters involved in these equations are defined in Table 1.

Equations A1, A3, and A4 consider the increase in the number of IL2 and mAbs in the blood due to the dissociation process of immune-complexes with a constant rate $\left(K_{\text{off}}^{\text{Ab}}\right)$, which corresponds to a decrease in the amount of these complexes (first term in Eqs A1, A3, and A4). The process of formation of immune-complexes, through the association of IL2 and mAb with a constant rate $\left(K_{\text{on}}^{\text{Ab}}\right)$, is taken into account in the second term in Eqs A1, A3, and A4. The exchange of molecules between blood and peripheral lymph nodes is modeled as a simple diffusion process that balances the molecule concentrations in both compartments (third term in Eqs A1, A3, and A4; first term in Eq. A2). The number of molecules decays exponentially with a constant rate (*K*_di_, *K*_da_), due to renal elimination in kidney (fourth term in Eqs A1, A3, and A4 and second term in Eq. A2). Finally, an external source for IL2, IL2m, and mAbs is considered, which causes an increase in the number of these molecules in the compartment (last term in Eqs A1–A3).

## Appendix B

### Dynamics of T cells in the lymph node compartment

The dynamics of the number of T cells in the lymph node compartment, following the process described above, are modeled with the following set of equations:

(B1) $$\begin{array}{llll} \frac{d\;E_{\text{N}}}{dt} & =\Gamma_{\text{e}}-K_{\text{A}}^{\text{E}}\times E_{\text{N}}^{\text{B}}\times {\left(1-\frac{R_{\text{T}}^{\text{B}}}{s\times A}\right)}^{\left(\text{s}-1\right)}\; & & \;\\ & \;+\alpha_{\text{E}}\times K_{\text{S}}^{\text{E}}\times \left(1-\frac{{\left(\text{SigE}\right)}^{\text{h}}}{{{(S}_{\text{E}})}^{\text{h}}+{\left(\text{SigE}\right)}^{\text{h}}}\right)\times E_{\text{A}}\; & & \;\\ & \;+\text{2}\;K_{\text{P}}^{\text{E}}\times E_{\text{C}}-K_{\text{d}}^{\text{E}}\times E_{\text{N}}^{\text{F}} \end{array}$$

(B2) $$\begin{array}{ll} \frac{d\;E_{\text{A}}}{dt} & =K_{\text{A}}^{\text{E}}\times E_{\text{N}}^{\text{B}}\times {\left(\text{1}-\frac{R_{\text{T}}^{\text{B}}}{s\times A}\right)}^{\left(\text{s}-1\right)}-K_{\text{S}}^{\text{E}}\times E_{\text{A}} \end{array}$$

(B3) $$\begin{array}{ll} \frac{d\;E_{\text{C}}}{dt} & =K_{\text{S}}^{\text{E}}\times \left(\frac{{\left(\text{SigE}\right)}^{\text{h}}}{{\left(S_{\text{E}}\right)}^{\text{h}}+{\left(\text{SigE}\right)}^{\text{h}}}\right)\times E_{\text{A}}-K_{\text{P}}^{\text{E}}\times E_{\text{C}} \end{array}$$

(B4) $$\begin{array}{llll} \frac{d\;R_{\text{N}}}{dt} & =\Gamma_{\text{r}}-K_{\text{A}}^{\text{R}}\times R_{\text{N}}^{\text{B}}+\alpha_{\text{R}}\;\times K_{\text{S}}^{\text{R}}\; & & \;\\ & \;\times \left(1-\frac{{\left(\text{SigR}\right)}^{\text{h}}}{{\left(S_{\text{R}}\right)}^{\text{h}}+{\left(\text{SigR}\right)}^{\text{h}}}\right)\times R_{\text{A}}+2\;K_{\text{P}}^{\text{R}}\; & & \;\\ & \;\times R_{\text{C}}-K_{\text{d}}^{\text{R}}\times R_{\text{N}}^{\text{F}} \end{array}$$

(B5) $$\begin{array}{ll} \frac{d\;R_{\text{A}}}{dt} & =K_{\text{A}}^{\text{R}}\times R_{\text{N}}^{\text{B}}-K_{\text{S}}^{\text{R}}\times R_{\text{A}} \end{array}$$

(B6) $$\begin{array}{llll} \frac{d\;R_{\text{C}}}{dt} & =K_{\text{S}}^{\text{R}}\times (\frac{{\left(\text{SigR}\right)}^{\text{h}}}{{\left(S_{\text{R}}\right)}^{\text{h}}+{\left(\text{SigR}\right)}^{\text{h}}})\; & & \;\\ & \;\times R_{\text{A}}-K_{\text{P}}^{\text{R}}\times R_{\text{C}} \end{array}$$

(B7) $$\begin{array}{llll} \frac{d\;M_{\text{A}}}{dt} & =-K_{\text{S}}^{\text{M}}\times (\frac{{\left(\text{SigM}\right)}^{\text{h}}}{{\left(S_{\text{M}}\right)}^{\text{h}}+{\left(\text{SigM}\right)}^{\text{h}}})\times M_{\text{A}}+\text{2}\; & & \;\\ & \;\times K_{\text{P}}^{\text{M}}\times M_{\text{C}}-K_{\text{d}}^{\text{M}}\times M_{\text{A}} \end{array}$$

(B8) $$\begin{array}{ll} \frac{d\;M_{\text{C}}}{dt} & =K_{\text{S}}^{\text{M}}\times (\frac{{\left(\text{SigM}\right)}^{\text{h}}}{{\left(S_{\text{M}}\right)}^{\text{h}}+{\left(\text{SigM}\right)}^{\text{h}}})\times M_{\text{A}}-K_{\text{P}}^{\text{M}}\times M_{\text{C}} \end{array}$$

(B9) $$\begin{array}{llll} \frac{K^{\text{E}}}{V_{\text{LN}}} & =E_{\text{l}}^{\text{B}}\text{/(}E_{\text{l}}^{\text{F}}\times F\text{);}\;\frac{K^{\text{R}}}{V_{\text{LN}}}=R_{\text{l}}^{\text{B}}∕\text{(}R_{\text{l}}^{\text{F}}\times F\text{)};\; & & \;\\ \frac{K^{\text{M}}}{V_{\text{LN}}} & =M_{\text{l}}^{\text{B}}∕\text{(}M_{\text{l}}^{\text{F}}\times F\text{)} \end{array}$$

(B10) $$\begin{array}{llll} \;F & =s\times A-\sum_{l}E_{1}^{\text{B}}-\sum_{l}R_{1}^{\text{B}}-\sum_{l}M_{1}^{\text{B}};\; & & \;\\ & \;\;\forall l\in \left\{\text{N},\;\text{A},\;\text{C}\right\} \end{array}$$

The dynamics of the number of Helper and Regulatory CD4^+^ T cells, on their three different functional states of their life cycle [resting (*E*_N_, *R*_N_), activated (*E*_A_, *R*_A_) and cycling (*E*_C_, *R*_C_) cells], is modeled using Eqs B1–B6, respectively; while the dynamics of the number of Memory CD8^+^ T cells on its two functional states [activated (*M*_A_) and cycling (*M*_C_) cells] is modeled with Eqs B7 and B8. The process of conjugation of T cells, on their different functional states, with their cognate APCs is modeled assuming quasi-steady state equilibrium, which leads to the equations presented in Eq. B9 [see a more detailed explanation of the derivation of these equations in (28)]. In Eq. B9, the symbolic label *l* denotes the functional state of the cell (*l* = *N*: resting, *l* = *A*: activated, *l* = *C*: cycling); and the superscript *B* and *F* denotes the cells conjugated to APC or free, respectively. The definitions of variables and parameters in Eqs B1–B9 are resumed in Table 2.

Equations B1–B6 considered that resting E and R cells are produced by the thymus (first term in Eqs B1 and B4), and they die with a constant rate $K_{\text{d}}^{\text{E}}\;\text{and}\;K_{\text{d}}^{\text{R}}$, respectively (last term in Eqs B1 and B4). Resting cells become activated after conjugation with APCs, process which is inhibited in E cells by the presence of co-conjugated R cells in the same APC (second term in Eqs B1 and B4; first term in Eqs B2 and B5). Activated T cells require enough cytokine derived signals to become cycling cells (first term in Eqs B3 and B6). The fraction of activated cells obtaining these signals is computed with a sigmoid function of the mean number of bound cytokines signaling receptors per cell (SigE, SigR). In the absence of these signals, a fraction α of the activated cells revert to the resting state (third term in Eqs B1 and B4) and the remaining fraction (1 − α) simply die. The cycling E and R cells divide producing two new resting cells with a constant rate $K_{\text{p}}^{\text{E}}\;\text{and}\;K_{\text{p}}^{\text{R}}$, respectively (fourth term in Eqs B1 and B4; second term in Eqs B3 and B6).

The Eqs B7 and B8 describe the dynamics of memory CD8^+^ T cells. In these equations, is modeled the dynamics of M cells analogous that for E cells. The only difference is that M cells are considered pre-activated cells, which become cycling in response to cytokine signals (first term in Eqs B7 and B8). The cycling M cells divide producing two new activated cells with a constant rate $K_{\text{p}}^{\text{M}}$ (second term in Eqs B7 and B8). The activated cells die with a constant rate $K_{\text{d}}^{\text{M}}$ (last term in Eq. B7).

## Appendix C

### Dynamics of molecules in the lymph node

The equations in the model describing the dynamics of the number of molecules circulating in the Lymph Node (IL2, anti-IL2 antibodies, and immune-complexes) and the number of complexes IL2-IL2R and IL2-mAb-IL2R formed in a single cell membrane are the following:

(C1) $$\begin{array}{llll} \frac{d\text{IL2}}{dt} & =K_{\text{off}}^{\text{Ab}}\times \text{IL}{\text{2}}^{\text{Ab}}-\frac{1}{N_{\text{A}}\times \text{fve}\times V_{\text{LN}}}K_{\text{on}}^{\text{Ab}}\times \text{IL2}\times \text{Ab}\; & & \;\\ & \;-\left(D_{\text{il}}\frac{\text{IL2}}{\text{fve}\times V_{\text{LN}}}-D_{\text{il}}\frac{\text{IL}{\text{2}}_{\text{S}}}{V_{\text{S}}}\right)+K_{\text{pi}}\times K_{\text{A}}^{\text{E}}\times E_{\text{N}}^{\text{B}}\; & & \;\\ & \;\times {\left(\text{1}-\frac{R_{\text{T}}^{\text{B}}}{s\times A}\right)}^{\left(s-1\right)}+\sum_{j}K_{\text{off}}^{\text{j}}\; & & \;\\ & \;\times \left[\;\sum_{l}\text{(}C_{\text{j}}^{{\text{E}}_{\text{l}}}\;\times E_{\text{l}}\text{)}\;+\sum_{l}(C_{j}^{{\text{R}}_{1}}\times R_{1})\;+\sum_{l}(C_{j}^{{\text{M}}_{1}}\times M_{1})\right]\; & & \;\\ & \;-\sum_{j}\frac{1}{N_{\text{A}}\times \text{fve}\times V_{\text{LN}}}K_{\text{on}}^{\text{j}}\times \text{IL2}\; & & \;\\ & \;\times \left[\;\sum_{l}\text{(}P_{\text{j}}^{{\text{E}}_{\text{l}}}\;\times E_{\text{l}}\text{)}\;+\sum_{l}\text{(}P_{\text{j}}^{{\text{R}}_{\text{l}}}\;\times R_{\text{l}}\text{)}\;+\sum_{l}(P_{j}^{{\text{M}}_{1}}\times M_{1})\right] \end{array}$$

(C2) $$\begin{array}{llll} \frac{d\text{IL2m}}{dt} & =-\left(D_{\text{il}}\frac{\text{IL2m}}{\text{fve}\times V_{\text{LN}}}-D_{\text{il}}\frac{\text{IL2}{\text{m}}_{\text{S}}}{V_{\text{S}}}\right)+\sum_{j}K_{\text{off}}^{\text{j}}\; & & \;\\ & \;\times \left[\sum_{l}{\text{(Cm}}_{\text{j}}^{{\text{E}}_{\text{l}}}\times E_{l}\text{)}+\sum_{l}{\text{(Cm}}_{\text{j}}^{{\text{R}}_{\text{l}}}\times R_{l}\text{)}\right.\; & & \;\\ & \;\left.+\sum_{l}{\text{(Cm}}_{\text{j}}^{{\text{M}}_{\text{l}}}\times M_{l}\text{)}\right]\; & & \;\\ & \;-\sum_{j}f_{j}\times \frac{1}{N_{\text{A}}\times \text{fve}\times V_{\text{LN}}}K_{\text{on}}^{\text{j}}\times \text{IL2m}\; & & \;\\ & \;\times \left[\;\sum_{l}\text{(}P_{\text{j}}^{{\text{E}}_{\text{l}}}\;\times E_{l}\text{)}\;+\sum_{l}\text{(}P_{\text{j}}^{{\text{R}}_{\text{l}}}\;\times R_{l}\text{)}\;+\sum_{l}\text{(}P_{\text{j}}^{{\text{M}}_{\text{l}}}\;\times M_{l}\text{)}\right] \end{array}$$

(C3) $$\begin{array}{llll} \frac{d\text{Ab}}{dt} & =K_{\text{off}}^{\text{Ab}}\times \text{IL}{\text{2}}^{\text{Ab}}-\frac{1}{N_{\text{A}}\times \text{fve}\times V_{\text{LN}}}K_{\text{on}}^{\text{Ab}}\; & & \;\\ & \;\times \text{IL2}\times \text{Ab}-\left(D_{\text{ab}}\frac{\text{Ab}}{\text{fve}\times V_{\text{LN}}}-D_{\text{ab}}\frac{\text{A}{\text{b}}_{\text{S}}}{V_{\text{S}}}\right)\; & & \;\\ & \;+\sum_{j}\text{(1}-N_{\text{j}}\text{)}\times \left[K_{\text{off}}^{\text{Ab}}\times \sum_{l}{\text{(CAb}}_{\text{j}}^{{\text{E}}_{\text{l}}}\times E_{\text{l}}\right.\; & & \;\\ & \;+{\text{CAb}}_{\text{j}}^{{\text{R}}_{\text{l}}}\times \left.R_{\text{l}}+{\text{CAb}}_{\text{j}}^{{\text{M}}_{\text{l}}}\times M_{\text{l}}\text{)}\right]\; & & \;\\ & \;-\sum_{j}\text{(1}-N_{\text{j}}\text{)}\times \left[\frac{1}{N_{\text{A}}\times \text{fve}\times V_{\text{LN}}}K_{\text{on}}^{\text{Ab}}\times \text{Ab}\right.\; & & \;\\ & \;\times \left.\sum_{l}\left(C_{\text{j}}^{{\text{E}}_{\text{l}}}\times E_{\text{l}}+C_{\text{j}}^{{\text{R}}_{\text{l}}}\times R_{\text{l}}+C_{\text{j}}^{{\text{M}}_{\text{l}}}\times M_{\text{l}}\right)\right] \end{array}$$

(C4) $$\begin{array}{llll} \frac{d\text{IL}{\text{2}}^{\text{Ab}}}{dt} & =-K_{\text{off}}^{\text{Ab}}\times \text{IL}{\text{2}}^{\text{Ab}}+\frac{1}{N_{\text{A}}\times \text{fve}\times V_{\text{LN}}}K_{\text{on}}^{\text{Ab}}\; & & \;\\ & \;\times \text{IL2}\times \text{Ab}-\left(D_{\text{ab}}\frac{\text{IL}{\text{2}}^{\text{Ab}}}{\text{fve}\times V_{\text{LN}}}-D_{\text{ab}}\frac{{\text{IL2}}_{\text{S}}^{\text{Ab}}}{V_{\text{S}}}\right)\; & & \;\\ & \;+\sum_{j}\text{(1}-N_{j}\text{)}\times K_{\text{off}}^{\text{j}}\; & & \;\\ & \;\times \left[\sum_{l}{\text{(CAb}}_{\text{j}}^{{\text{E}}_{\text{l}}}\times E_{l}\text{)}+\sum_{l}{\text{(CAb}}_{\text{j}}^{{\text{R}}_{\text{l}}}\times R_{l}\text{)}\right.\; & & \;\\ & \;+\left.\sum_{l}{\text{(CAb}}_{\text{j}}^{{\text{M}}_{\text{l}}}\times M_{l}\text{)}\right]-\sum_{j}\text{(1}-N_{j}\text{)}\; & & \;\\ & \;\times \frac{1}{N_{\text{A}}\times \text{fve}\times V_{\text{LN}}}K_{\text{on}}^{\text{j}}\times \text{IL}{\text{2}}^{\text{Ab}}\; & & \;\\ & \;\times \left[\sum_{l}\text{(}P_{\text{j}}^{{\text{E}}_{\text{l}}}\times E_{l}\text{)}+\sum_{l}\text{(}P_{\text{j}}^{{\text{R}}_{\text{l}}}\times R_{l}\text{)}\right.\; & & \;\\ & \;+\left.\sum_{l}\text{(}P_{\text{j}}^{{\text{M}}_{\text{l}}}\times M_{l}\text{)}\right] \end{array}$$

(C5) $$\begin{array}{llll} \frac{d{\text{Cm}}_{\alpha }^{{\text{E}}_{\text{l}}}}{dt} & =f_{\alpha }\times \;K_{\text{on}}^{\alpha }\times \frac{1}{N_{\text{A}}\times \text{fve}\times V_{\text{LN}}}\; & & \;\\ & \;\times \text{IL2m}\times P_{\alpha }^{{\text{E}}_{\text{l}}}-K_{\text{off}}^{\alpha }\times {\text{Cm}}_{\alpha }^{{\text{E}}_{\text{l}}}-f_{\beta }\times K_{\text{on}}^{\alpha \beta }\; & & \;\\ & \;\times {\text{Cm}}_{\alpha }^{{\text{E}}_{\text{l}}}\times P_{\beta }^{{\text{E}}_{\text{l}}}+K_{\text{off}}^{\alpha \beta }\times \text{T}{\text{m}}^{{\text{E}}_{\text{l}}} \end{array}$$

(C6) $$\begin{array}{llll} \frac{d{\text{Cm}}_{\beta }^{{\text{E}}_{\text{l}}}}{dt} & =f_{\beta }\times K_{\text{on}}^{\beta }\times \frac{1}{N_{\text{A}}\times \text{fve}\times V_{\text{LN}}}\; & & \;\\ & \;\times \text{IL2m}\times P_{\beta }^{{\text{E}}_{\text{l}}}-K_{\text{off}}^{\beta }\times {\text{Cm}}_{\beta }^{{\text{E}}_{\text{l}}}-f_{\alpha }\times K_{\text{on}}^{\beta \alpha }\; & & \;\\ & \;\times {\text{Cm}}_{\beta }^{{\text{E}}_{\text{l}}}\times P_{\alpha }^{{\text{E}}_{\text{l}}}+K_{\text{off}}^{\beta \alpha }\times \text{T}{\text{m}}^{{\text{E}}_{\text{l}}}-K_{\text{in}}\times {\text{Cm}}_{\beta }^{{\text{E}}_{\text{l}}} \end{array}$$

(C7) $$\begin{array}{llll} \frac{d\text{T}{\text{m}}^{{\text{E}}_{\text{l}}}}{dt} & =f_{\beta }\times K_{\text{on}}^{\alpha \beta }\times {\text{Cm}}_{\alpha }^{{\text{E}}_{\text{l}}}\times P_{\beta }^{{\text{E}}_{\text{l}}}-K_{\text{off}}^{\alpha \beta }\times \text{T}{\text{m}}^{{\text{E}}_{\text{l}}}\; & & \;\\ & \;+f_{\alpha }\times K_{\text{on}}^{\beta \alpha }\times {\text{Cm}}_{\beta }^{{\text{E}}_{\text{l}}}\times P_{\alpha }^{{\text{E}}_{\text{l}}}-K_{\text{off}}^{\beta \alpha }\times \text{T}{\text{m}}^{{\text{E}}_{\text{l}}}\; & & \;\\ & \;-K_{\text{in}}\times \text{T}{\text{m}}^{{\text{E}}_{\text{l}}} \end{array}$$

(C8) $$\begin{array}{llll} \frac{d{\text{C}}_{\alpha }^{{\text{E}}_{\text{l}}}}{dt} & =K_{\text{on}}^{\alpha }\times \frac{1}{N_{\text{A}}\times \text{fve}\times V_{\text{LN}}}\times \text{IL2}\times P_{\alpha }^{{\text{E}}_{\text{l}}}-K_{\text{off}}^{\alpha }\; & & \;\\ & \;\times C_{\alpha }^{{\text{E}}_{\text{l}}}-K_{\text{on}}^{\alpha \beta }\times C_{\alpha }^{{\text{E}}_{\text{l}}}\times P_{\beta }^{{\text{E}}_{\text{l}}}+K_{\text{off}}^{\alpha \beta }\times T^{{\text{E}}_{\text{l}}}\; & & \;\\ & \;+\text{(1}-N_{\alpha }\text{)}\times \left(-\frac{1}{N_{\text{A}}\times \text{fve}\times V_{\text{LN}}}K_{\text{on}}^{\text{Ab}}\right.\; & & \;\\ & \;\left.\times C_{\alpha }^{{\text{E}}_{\text{l}}}\times \text{Ab}+K_{\text{off}}^{\text{Ab}}\times {\text{CAb}}_{\alpha }^{{\text{E}}_{\text{l}}}\right) \end{array}$$

(C9) $$\begin{array}{llll} \frac{dC_{\beta }^{E_{l}}}{dt} & =K_{on}^{\beta }\times \frac{1}{N_{\text{A}}\times \text{fve}\times V_{\text{LN}}}\times \text{IL2}\times P_{\beta }^{{\text{E}}_{\text{l}}}-K_{\text{off}}^{\beta }\; & & \;\\ & \;\times C_{\beta }^{{\text{E}}_{\text{l}}}-K_{\text{on}}^{\beta \alpha }\times C_{\beta }^{{\text{E}}_{\text{l}}}\times P_{\alpha }^{{\text{E}}_{\text{l}}}+K_{\text{off}}^{\beta \alpha }\times T^{{\text{E}}_{\text{l}}}\; & & \;\\ & \;+\text{(1}-N_{\beta }\text{)}\times \left(-\frac{1}{N_{\text{A}}\times \text{fve}\times V_{\text{LN}}}K_{\text{on}}^{\text{Ab}}\right.\; & & \;\\ & \;\left.\times C_{\beta }^{{\text{E}}_{\text{l}}}\times \text{Ab}+K_{\text{off}}^{\text{Ab}}\times {\text{CAb}}_{\beta }^{{\text{E}}_{\text{l}}}\right)-K_{\text{in}}\times C_{\beta }^{{\text{E}}_{\text{l}}} \end{array}$$

(C10) $$\begin{array}{llll} \frac{dT^{{\text{E}}_{\text{l}}}}{dt} & =K_{\text{on}}^{\alpha \beta }\times C_{\alpha }^{{\text{E}}_{\text{l}}}\times P_{\beta }^{{\text{E}}_{\text{l}}}-K_{\text{off}}^{\alpha \beta }\times T^{{\text{E}}_{\text{l}}}+K_{\text{on}}^{\beta \alpha }\times C_{\beta }^{{\text{E}}_{\text{l}}}\; & & \;\\ & \;\times P_{\alpha }^{{\text{E}}_{\text{l}}}-K_{\text{off}}^{\beta \alpha }\times T^{{\text{E}}_{\text{l}}}-K_{\text{in}}\times T^{{\text{E}}_{\text{l}}} \end{array}$$

(C11) $$\begin{array}{llll} \frac{d{\text{CAb}}_{\alpha }^{{\text{E}}_{\text{l}}}}{dt} & =\text{(1}-N_{\alpha }\text{)}\times \left(K_{\text{on}}^{\alpha }\times \frac{1}{N_{\text{A}}\times \text{fve}\times V_{\text{LN}}}\times \text{IL}{\text{2}}^{\text{Ab}}\right.\; & & \;\\ & \;\times \left.P_{\alpha }^{{\text{E}}_{\text{l}}}-K_{\text{off}}^{\alpha }\times {\text{CAb}}_{\alpha }^{{\text{E}}_{\text{l}}}\right)+\text{(1}-N_{\alpha }\text{)}\; & & \;\\ & \;\times \left(\frac{1}{N_{\text{A}}\times \text{fve}\times V_{\text{LN}}}K_{\text{on}}^{\text{Ab}}\times C_{\alpha }^{{\text{E}}_{\text{l}}}\times \text{Ab}\right.\; & & \;\\ & \;\left.-K_{\text{off}}^{\text{Ab}}\times {\text{CAb}}_{\alpha }^{{\text{E}}_{\text{l}}}\right) \end{array}$$

(C12) $$\begin{array}{llll} \frac{d{\text{CAb}}_{\beta }^{{\text{E}}_{\text{l}}}}{dt} & =\text{(1}-N_{\beta }\text{)}\times \left(K_{\text{on}}^{\beta }\times \frac{1}{N_{\text{A}}\times \text{fve}\times V_{\text{LN}}}\right.\; & & \;\\ & \;\left.\times \text{IL}{\text{2}}^{\text{Ab}}\times P_{\beta }^{{\text{E}}_{\text{l}}}-K_{\text{off}}^{\beta }\times {\text{CAb}}_{\beta }^{{\text{E}}_{\text{l}}}\right)+\text{(1}-N_{\beta }\text{)}\; & & \;\\ & \;\times \left(\frac{1}{N_{\text{A}}\times \text{fve}\times V_{\text{LN}}}K_{\text{on}}^{\text{Ab}}\times C_{\beta }^{{\text{E}}_{\text{l}}}\times \text{Ab}\right.\; & & \;\\ & \;\left.-K_{\text{off}}^{\text{Ab}}\times {\text{CAb}}_{\beta }^{{\text{E}}_{\text{l}}}\right)\; & & \;\\ & \;-K_{\text{in}}\times {\text{CAb}}_{\beta }^{{\text{E}}_{\text{l}}} \end{array}$$

(C13) $$\begin{array}{llll} P_{\alpha }^{{\text{E}}_{\text{l}}} & =\text{R}{\text{a}}^{{\text{E}}_{\text{l}}}-C_{\alpha }^{{\text{E}}_{\text{l}}}-{\text{CAb}}_{\alpha }^{{\text{E}}_{\text{l}}}-T^{{\text{E}}_{\text{l}}}-{\text{Cm}}_{\alpha }^{{\text{E}}_{\text{l}}}-\text{T}{\text{m}}^{{\text{E}}_{\text{l}}},\; & & \;\\ P_{\beta }^{{\text{E}}_{\text{l}}} & =\text{R}{\text{b}}^{{\text{E}}_{\text{l}}}-C_{\beta }^{{\text{E}}_{\text{l}}}-{\text{CAb}}_{\beta }^{{\text{E}}_{\text{l}}}-T^{{\text{E}}_{\text{l}}}-{\text{Cm}}_{\beta }^{{\text{E}}_{\text{l}}}-\text{T}{\text{m}}^{{\text{E}}_{\text{l}}} \end{array}$$

(C14) $$\begin{array}{llll} \text{SigE} & =C_{\beta }^{E_{A}}+T^{E_{A}}+{\text{Cm}}_{\beta }^{E_{A}}+{\text{Tm}}^{E_{A}}+{\text{CAb}}_{\beta }^{E_{A}}+\text{il}\alpha_{E_{A}},\; & & \;\\ \text{SigR} & =C_{\beta }^{R_{A}}+T^{R_{A}}+{\text{Cm}}_{\beta }^{R_{A}}+{\text{Tm}}^{R_{A}}+{\text{CAb}}_{\beta }^{R_{A}},\\ \text{SigM} & =C_{\beta }^{M_{A}}+T^{M_{A}}+{\text{Cm}}_{\beta }^{M_{A}}+{\text{Tm}}^{M_{A}}+{\text{CAb}}_{\beta }^{M_{A}}+\text{il}\alpha_{M_{A}}\; & & \; \end{array}$$

The dynamics of the number of IL2 (IL2), IL2 mutants (IL2m), mAbs (Ab), and immune-complexes (IL2^Ab^) in the lymph node is modeled using Eqs C1 and C4; while the dynamics of the number of IL2-IL2R complexes $\left(C_{\alpha }^{\text{E}},\;C_{\beta }^{\text{E}},\;T^{\text{E}}\right)$; IL2m-IL2R complexes $\left({\text{Cm}}_{\alpha }^{\text{E}},\;{\text{Cm}}_{\beta }^{\text{E}},\;{\text{Tm}}^{\text{E}}\right)$; and IL2-IL2R-mAbs complexes ${\text{CAb}}_{\alpha }^{\text{E}},{\text{CAb}}_{\beta }^{\text{E}}$ per cell are modeled following equations (22–24); (19–21); and (25, 26), respectively. Note that, to simplify, we only present here the equations corresponding to the IL2 and IL2m complexes formed at the E cell membrane. Equivalent equations are written for R and M cells. Algebraic relations are provided in Eqs C13 and C14, for the amount of free alpha $\left(P_{\alpha }^{\text{E}}\right)$ and beta chains $\left(P_{\beta }^{\text{E}}\right)$ of the IL2 receptor per E cell, and the mean number of bound cytokines signaling receptors per activated E (SigE), R (SigR), and M (SigM) cell. Note that, the terms SigE, SigR, and SigM in Eq. C14 are the one used in the equations for the dynamics of T cells, related with the process of cells receiving cytokine derived signals from IL2 or alternative cytokines (see section 1.2). The variables and parameters used in Ref. (15–28) are defined in Tables 1–3.

In Eqs C1, C3, and C4, the first and second terms correspond to the processes of dissociation and formation of immune-complexes. Additionally, due to the presence of T cells in the Lymph Node, we consider in these equations the dissociation and association processes of IL2 and IL2-mAb complexes with the alpha or beta IL2R chains in the cell membranes. In this sense, is taken into account the increase in the amount of free IL2, IL2m, and immune-complexes, due to the dissociation process of IL2-IL2R, IL2m-IL2R, and IL2-mAb-IL2R complexes respectively (third terms in Eqs C1 and C4 and second term in Eq. C2). On the other hand, the association of free IL2, IL2m, and immune-complexes to free alpha or beta IL2R chains is considered to increase the amount of IL2-IL2R and IL2-mAb-IL2R complexes (fourth terms in Eqs C1 and C4, third term in Eq. C2). Additionally, is modeled the processes where mAbs can be dissociated from IL2-mAb-IL2R complexes, increasing the number of free mAbs (third terms in Eq. C3); and the process where free mAbs associate to IL2-IL2R complexes in the cell membrane (fourth terms in Eq. C3). The symbolic labels *l* and *j*, appearing in the equations (15–18), denote respectively the functional state of the cell (*l* = *N*: resting, *l* = *A*: activated, *l* = *C*: cycling) and the different IL2R chains (*j* = α alpha chain and *j* = β beta dimer chain). Finally, the production of IL2 endogenous by activated E cells, which can be inhibited during cell activation by the presence of R cells co-conjugated in the same APC, is considered to increase the amount of this cytokine in the Lymph Node (last term in Eq. C1). The properties of different IL2m is controlled in the model by the parameters *f*_α_ and *f*_β_ which multiply the association of constant of this molecules to the alpha and beta chain of the IL2 receptor.

The formation of high affinity IL2-IL2R and IL2m-IL2R complexes in a cell membrane is modeled as a two-step process, using equations (22–24) and (19–21) respectively. Firstly, free IL2 or IL2m binds to the available free alpha or beta chains of the IL2R, forming the intermediate or low affinity IL2-IL2R complexes respectively (first terms in Eqs C8, C9 and C5, C6), as mentioned above for the dynamics of IL2. By the corresponding dissociation process are recovered free molecules and receptor chains (second term in Eqs C8, C9 and C5, C6). The association process of intermediate or low affinity IL2-IL2R complexes with the remaining IL2 receptor chain, leads to the formation of high affinity IL2-IL2R complexes (third term in Eqs C8, C9 and C5, C6), and first and third terms in Eqs C10 and C7. The dissociation of these complexes is modeled in the fourth term in Eqs C9, C10 and C5, C6 and second and fourth terms in Eqs C10 and C7. The internalization of IL2 and IL2m forming complexes with IL2Rs is modeled considering that it only occurs for signaling IL2-IL2R complexes requiring binding to the beta chain (last term in Eqs C9, C10 and C6, C7).

The formation of IL2-mAb-IL2R complexes in the cell membrane is modeled in Eqs C11 and C12, and in the fifth term in Eqs C8 and C9. In this sense, we consider the association and dissociation processes of free immune-complexes with the alpha or beta IL2R chains (first term in Eqs C11 and C12); and the same processes for free mAbs with IL2-IL2R complexes in the cell membrane (fifth term in Eqs C8 and C9); second term in Eqs C11 and C12). The possibility of formation of intermediate or low affinity IL2-mAb-IL2R complexes depend on the IL2 interface that mAbs recognize (controlled in simulations by the parameter *N_j_*, see Table 3). We don’t consider the formation of high affinity IL2-mAb-IL2R complexes, due to association of antibodies with the high affinity IL2-IL2R complexes or the association of intermediate or low affinity IL2-mAb-IL2R complexes with the remaining IL2 receptor chain, because we are studying mAbs that bind to the alpha or beta interface of the IL2 which will block the formation of these complexes. Finally, the internalization of IL2 as immune-complexes bound to the beta chains of IL2Rs in the cell membrane is also modeled (last term in Eq. C12).

## References

[1] Kudo-Saito C Garnett CT Wansley EK Schlom J Hodge JW Intratumoral delivery of vector mediated IL-2 in combination with vaccine results in enhanced T cell avidity and anti-tumor activity. Cancer Immunol Immunother (2007) 56(12):1897–910 10.1007/s00262-007-0332-1 17503041 PMC11030948

[2] Fishman M Hunter TB Soliman H Thompson P Dunn M Smilee R Phase II trial of B7-1 (CD-86) transduced, cultured autologous tumor cell vaccine plus subcutaneous interleukin-2 for treatment of stage IV renal cell carcinoma. J Immunother (2008) 31(1):72–80 10.1097/CJI.0b013e31815ba792 18157014

[3] Lin CT Tsai YC He L Yeh CN Chang TC Soong YK DNA vaccines encoding IL-2 linked to HPV-16 E7 antigen generate enhanced E7-specific CTL responses and antitumor activity. Immunol Lett (2007) 114(2):86–93 10.1016/j.imlet.2007.09.008 17976741 PMC2169502

[4] Tarpey I van Loon AA de Haas N Davis PJ Orbell S Cavanagh D A recombinant turkey Herpesvirus expressing chicken interleukin-2 increases the protection provided by in ovo vaccination with infectious bursal disease and infectious bronchitis virus. Vaccine (2007) 25(51):8529–35 10.1016/j.vaccine.2007.10.006 17996994

[5] Davey RT Jr Murphy RL Graziano FM Boswell SL Pavia AT Cancio M Immunologic and virologic effects of subcutaneous interleukin 2 in combination with antiretroviral therapy: a randomized controlled trial. JAMA (2000) 284(2):183–9 10.1001/jama.284.2.183 10889591

[6] Kovacs JA Vogel S Albert JM Falloon J Davey RT Jr Walker RE Controlled trial of interleukin-2 infusions in patients infected with the human immunodeficiency virus. N Engl J Med (1996) 335(18):1350–6 10.1056/NEJM199610313351803 8857018

[7] Sereti I Martinez-Wilson H Metcalf JA Baseler MW Hallahan CW Hahn B Long-term effects of intermittent interleukin 2 therapy in patients with HIV infection: characterization of a novel subset of CD4(+)/CD25(+) T cells. Blood (2002) 100(6):2159–67 12200381

[8] Natarajan V Lempicki RA Sereti I Badralmaa Y Adelsberger JW Metcalf JA Increased peripheral expansion of naive CD4+ T cells in vivo after IL-2 treatment of patients with HIV infection. Proc Natl Acad Sci U S A (2002) 99(16):10712–7 10.1073/pnas.162352399 12149467 PMC125022

[9] Ahmadzadeh M Rosenberg SA IL-2 administration increases CD4^+^ CD25(hi) Foxp3^+^ regulatory T cells in cancer patients. Blood (2006) 107(6):2409–14 10.1182/blood-2005-06-2399 16304057 PMC1473973

[10] Montero E Alonso L Perez R Lage A Interleukin-2 mastering regulation in cancer and autoimmunity. Ann N Y Acad Sci (2007) 1107:239–50 10.1196/annals.1381.026 17804552

[11] Tang Q Adams JY Penaranda C Melli K Piaggio E Sgouroudis E Central role of defective interleukin-2 production in the triggering of islet autoimmune destruction. Immunity (2008) 28(5):687–97 10.1016/j.immuni.2008.03.016 18468463 PMC2394854

[12] Grinberg-Bleyer Y Baeyens A You S Elhage R Fourcade G Gregoire S IL-2 reverses established type 1 diabetes in NOD mice by a local effect on pancreatic regulatory T cells. J Exp Med (2010) 207(9):1871–8 10.1084/jem.20100209 20679400 PMC2931175

[13] Setoguchi R Hori S Takahashi T Sakaguchi S Homeostatic maintenance of natural Foxp3(+) CD25(+) CD4(+) regulatory T cells by interleukin (IL)-2 and induction of autoimmune disease by IL-2 neutralization. J Exp Med (2005) 201(5):723–35 10.1084/jem.20041982 15753206 PMC2212841

[14] Onizuka S Tawara I Shimizu J Sakaguchi S Fujita T Nakayama E Tumor rejection by in vivo administration of anti-CD25 (interleukin-2 receptor alpha) monoclonal antibody. Cancer Res (1999) 59(13):3128–33 10397255

[15] Church AC Clinical advances in therapies targeting the interleukin-2 receptor. QJM (2003) 96(2):91–102 10.1093/qjmed/hcg014 12589007

[16] Kamimura D Sawa Y Sato M Agung E Hirano T Murakami M IL-2 in vivo activities and antitumor efficacy enhanced by an anti-IL-2 mAb. J Immunol (2006) 177(1):306–14 16785526 10.4049/jimmunol.177.1.306

[17] Mostbock S Lutsiak ME Milenic DE Baidoo K Schlom J Sabzevari H IL-2/anti-IL-2 antibody complex enhances vaccine-mediated antigen-specific CD8(+) T cell responses and increases the ratio of effector/memory CD8(+) T cells to regulatory T cells. J Immunol (2008) 180(7):5118–29 18354238 10.4049/jimmunol.180.7.5118

[18] Webster KE Walters S Kohler RE Mrkvan T Boyman O Surh CD In vivo expansion of Treg cells with IL-2-mAb complexes: induction of resistance to EAE and long-term acceptance of isletallografts without immunosuppression. J Exp Med (2009) 206(4):751–60 10.1084/jem.20082824 19332874 PMC2715127

[19] Boyman O Surh CD Sprent J Potential use of IL-2/anti-IL-2 antibody immune complexes for the treatment of cancer and autoimmune disease. Expert Opin Biol Ther (2006) 6(12):1323–31 10.1517/14712598.6.12.1323 17223740

[20] Boyman O Kovar M Rubinstein MP Surh CD Sprent J Selective stimulation of T cell subsets with antibody-cytokine immune complexes. Science (2006) 311:1924–7 10.1126/science.1122927 16484453

[21] Smith KA Interleukin-2: inception, impact, and implications. Science (1988) 240(4856):1169–76 10.1126/science.3131876 3131876

[22] Almeida AR Legrand N Papiernik M Freitas AA Homeostasis of peripheral CD4^+^ T cells: IL-2R alpha and IL-2 shape a population of regulatory cells that controls CD4+ T cell numbers. J Immunol (2002) 169(9):4850–60 12391195 10.4049/jimmunol.169.9.4850

[23] Grinberg-Bleyer Y Saadoun D Baeyens A Billiard F Goldstein JD Grégoire S Pathogenic T cells have a paradoxical protective effect in murine autoimmune diabetes by boosting Tregs. J Clin Invest (2010) 120(12):4558–68 10.1172/JCI42945 21099113 PMC2993590

[24] Almeida AR Zaragoza B Freitas AA Indexation as a novel mechanism of lymphocyte homeostasis: the number of CD4+CD25+ regulatory T cells is indexed to the number of IL-2-producing cells. J Immunol (2006) 177(1):192–200 16785514 10.4049/jimmunol.177.1.192

[25] Barthlott T Moncrieffe H Veldhoen M Atkins CJ Christensen J O’Garra A CD25+ CD4+ T cells compete with naive CD4+ T cells for IL-2 and exploit it for the induction of IL-10 production. Int Immunol (2005) 17(3):279–88 10.1093/intimm/dxh207 15684039

[26] Thornton AM Shevach EM CD4+CD25+ immunoregulatory T cells suppress polyclonal T cell activation in vitro by inhibiting interleukin 2 production. J Exp Med (1998) 188(2):287–96 10.1084/jem.188.2.287 9670041 PMC2212461

[27] Garcia-Martinez K Leon K Modeling the role of IL-2 in the interplay between CD4+ helper and regulatory T cells: studying the impact of IL2 modulation therapies. Int Immunol (2012) 24(7):427–46 10.1093/intimm/dxr120 22371423

[28] Garcia-Martinez K Leon K Modeling the role of IL-2 in the interplay between CD4+ helper and regulatory T cells: assessing general dynamical properties. J Theor Biol (2010) 262(4):720–32 10.1016/j.jtbi.2009.10.025 19878686

[29] Smith KA The structure of IL2 bound to the three chains of the IL2 receptor and how signaling occurs. Med Immunol (2006) 5:3 10.1186/1476-9433-5-3 16907989 PMC1562422

[30] Smith KA The quantal theory of how the immune system discriminates between “self and non-self.” Med Immunol (2004) 3(1):3 10.1186/1476-9433-3-3 15606917 PMC544850

[31] Kuniyasu Y Takahashi T Itoh M Shimizu J Toda G Sakaguchi S Naturally anergic and suppressive CD25(+)CD4(+) T cells as a functionally and phenotypically distinct immunoregulatory T cell subpopulation. Int Immunol (2000) 12(8):1145–55 10.1093/intimm/12.8.1145 10917889

[32] Létourneau S Krieg C Pantaleo G Boyman O IL-2- and CD25-dependent immunoregulatory mechanisms in the homeostasis of T-cell subsets. J Allergy Clin Immunol (2009) 123(4):758–62 10.1016/j.jaci.2009.02.011 19348914

[33] León K Peréz R Lage A Carneiro J Modelling T-cell-mediated suppression dependent on interactions in multicellular conjugates. J Theor Biol (2000) 207(2):231–54 10.1006/jtbi.2000.2169 11034831

[34] Carneiro J Leon K Caramalho I van denDoolC Gardner R Oliveira V When three is not a crowd: a crossregulation model of the dynamics and repertoire selection of regulatory CD4+ T cells. Immunol Rev (2007) 216:48–68 17367334 10.1111/j.1600-065X.2007.00487.x

[35] Blattman JN Grayson JM Wherry EJ Kaech SM Smith KA Ahmed R Therapeutic use of IL-2 to enhance antiviral T-cell responses in vivo. Nat Med (2003) 9(5):540–7 10.1038/nm866 12692546

[36] Cesana GC DeRaffele G Cohen S Moroziewicz D Mitcham J Stoutenburg J Characterization of CD4+CD25+ regulatory T cells in patients treated with high-dose interleukin-2 for metastatic melanoma or renal cell carcinoma. J Clin Oncol (2006) 24(7):1169–77 10.1200/JCO.2005.03.6830 16505437

[37] Sereti I Imamichi H Natarajan V Imamichi T Ramchandani MS Badralmaa Y In vivo expansion of CD4CD45RO-CD25 T cells expressing foxP3 in IL-2-treated HIV-infected patients. J Clin Invest (2005) 115(7):1839–47 10.1172/JCI24307 15937547 PMC1142113

[38] Rojas G Pupo A Leon K Avellanet J Carmenate T Sidhu S Deciphering the molecular bases of the biological effects of antibodies against Interleukin-2: a versatile platform for fine epitope mapping. Immunobiology (2013) 218(1):105–13 10.1016/j.imbio.2012.02.009 22459271

[39] Létourneau S van Leeuwen EM Krieg C Martin C Pantaleo G Sprent J IL-2/anti-IL-2 antibody complexes show strong biological activity by avoiding interaction with IL-2 receptor alpha subunit CD25. Proc Natl Acad Sci U S A (2010) 107(5):2171–6 10.1073/pnas.0909384107 20133862 PMC2836659

[40] Carmenate T Pacios A Enamorado M Moreno E Garcia-Martínez K Fuente D Human IL-2 mutein with higher antitumor efficacy than wild type IL-2. J Immunol (2013) 190(12):6230–8 10.4049/jimmunol.1201895 23677467

[41] Rao BM Driver I Lauffenburger DA Wittrup KD High-affinity CD25-binding IL-2 mutants potently stimulate persistent T cell growth. Biochemistry (2005) 44(31):10696–701 10.1021/bi050436x 16060678

[42] Levin AM Bates DL Ring AM Krieg C Lin JT Su L Exploiting a natural conformational switch to engineer an interleukin-2 ‘superkine.’ Nature (2012) 484(7395):529–33 10.1038/nature10975 22446627 PMC3338870

[43] Harvill ET Fleming JM Morrison SL In vivo properties of an IgG3-IL-2 fusion protein. A general strategy for immune potentiation. J Immunol (1996) 157(7):3165–70 8816429

[44] Williams MA Tyznik AJ Bevan MJ Interleukin-2 signals during priming are required for secondary expansion of CD8+ memory T cells. Nature (2006) 441(7095):890–3 10.1038/nature04790 16778891 PMC2776073

[45] Kamimura D Bevan MJ Naive CD8+ T cells differentiate into protective memory-like cells after IL-2 anti IL-2 complex treatment in vivo. J Exp Med (2007) 204(8):1803–12 10.1084/jem.20070543 17664293 PMC2118678

[46] Zheng SG Wang J Wang P Gray JD Horwitz DA IL-2 is essential for TGF-beta to convert naive CD4+CD25− cells to CD25+Foxp3+ regulatory T cells and for expansion of these cells. J Immunol (2007) 178(4):2018–27 17277105 10.4049/jimmunol.178.4.2018

[47] Krieg C Letourneau S Pantaleo G Boyman O Improved IL-2 immunotherapy by selective stimulation of IL-2 receptors on lymphocytes and endothelial cells. Proc Natl Acad Sci U S A (2010) 107:11906 10.1073/pnas.1002569107 20547866 PMC2900642

[48] Wolf M Schimpl A Hunig T Control of T cell hyperactivation in IL-2-deficient mice by CD4(+)CD25(−) and CD4(+)CD25(+) T cells: evidence for two distinct regulatory mechanisms. Eur J Immunol (2001) 31(6):1637–45 10.1002/1521-4141(200106)31:6<1637::AID-IMMU1637>3.0.CO;2-T 11385607

[49] Malek TR Porter BO Codias EK Scibelli P Yu A Normal lymphoid homeostasis and lack of lethal autoimmunity in mice containing mature T cells with severely impaired IL-2 receptors. J Immunol (2000) 164(6):2905–14 10706676 10.4049/jimmunol.164.6.2905

